# Low-dose gamma irradiation enhances the enzymatic ochratoxin A detoxification potential of a soil-derived *Bacillus thuringiensis*

**DOI:** 10.3389/fmicb.2026.1837344

**Published:** 2026-05-14

**Authors:** Eslam T. Mohamed, Nashwa H. Abdullah, Khaloud Mohammed Alarjani, Mohamed E. Osman, Shaimaa Abd El Mohsen Ibrahim, Eman Tawfik

**Affiliations:** 1 Botany and Microbiology Department, Faculty of Science, Capital University (formerly Helwan University), Cairo, Egypt; 2 Department of Botany and Microbiology, College of Science, King Saud University, Riyadh, Saudi Arabia; 3 Radiation Microbiology Department, National Center for Radiation Research and Technology (NCRRT), Egyptian Atomic Energy Authority (EAEA), Cairo, Egypt

**Keywords:** *Aspergillus niger*, *Bacillus thuringiensis*, biocontrol, detoxification, gamma radiation, molecular docking, ochratoxin A

## Abstract

Contamination of food by *Aspergillus niger* is a serious global food safety issue due to ochratoxin A (OTA). This study evaluates the use of low-dose gamma irradiation to maximize the mycotoxin-detoxifying properties of a native *Bacillus thuringiensis* biocontrol agent isolated from soil. A soil-derived *B. thuringiensis* strain demonstrated significant intrinsic antifungal activity against an OTA-producing *A. niger* isolate, which was correlated with the production of chitinases, glucanases, and proteases. To maximize this biocontrol potential, gamma irradiation of bacterial cell-free filtrates was performed. A 0.2 kGy dose of radiation optimally increased the bioactivity of the filtrate, increasing the inhibition of *A. niger* radial growth (from 82.8 to 88.3% compared to the untreated control) and the enzymatic breakdown of OTA into the less toxic ochratoxin α (OTα) in both liquid cultures and contaminated wheat grains. Enzymatic activity tests showed that the 0.2 kGy irradiation exposure produced an 18.06% increase in carboxypeptidase activity. Moreover, FTIR analysis and *in silico* molecular docking were used to provide supportive mechanistic information about the increased activity observed following irradiation. The findings indicate that the structural alterations caused by mild radiation stimulated enzyme-substrate interactions and increased the binding affinity of the carboxypeptidase enzyme to OTA, without destroying its structural scaffold. These results indicate that low-dose gamma irradiation can be an extremely useful method for enzymatic activity improvement, which can be considered a promising and feasible biotechnological solution for mycotoxin mitigation in agricultural matrices.

## Introduction

*Aspergillus niger* produces mycotoxins, the most hazardous of which is ochratoxin A (OTA), a recalcitrant, highly toxic chemical that is a major threat to global food safety and requires effective approaches based on microbial biodegradation (Zou et al., 2022; Ismaiel et al., 2023). Conventional chemical and physical means of mitigation are not always effective, necessitating new biotechnological processes that use active microbial biocatalysts to treat environmental and food matrices (Rawat et al., 2024; Abbasi, 2025). Moreover, mycotoxins are linked to serious adverse health complications because of the buildup of these harmful fungal compounds in food matrices (Balendres et al., 2019). Mycotoxins have been reported to induce severe health conditions such as mutagenic, carcinogenic, immunosuppressive, neurotoxic, and estrogenic diseases (Fakhri et al., 2025).

*Aspergillus niger* is a common mycotoxigenic fungus that is a notorious mycotoxin-producing species, particularly ochratoxin A (OTA) which is a potent nephrotoxin with carcinogenic properties (Gurikar et al., 2023). The prevalence of *A. niger* is significantly high in warm and humid regions such as countries of North Africa. Egypt has been reported as a hotspot for OTA contamination in wheat crops. The isolation of OTA-producing *A. niger* strains from local Egyptian grain highlights the urgent need for effective, safe, and sustainable control strategies (Awaad et al., 2021).

A range of physical and chemical techniques have been employed as a preventive procedure for mycotoxin control such as the use of fungicides, adsorbents, and other chemical treatments. However, these methods did not achieve satisfactory results as they often affect the quality of the processed food or lead to other associated health issues. Recently, focus has been shifted to biological approaches as innovative mycotoxin control methods that can be employed as smart alternatives to these conventional strategies due to their safety, low cost and higher efficiency. Moreover, this approach can play a role in improving food quality by reducing the load of chemical residues on food materials that resulted from the chemical control methods (Nguyen et al., 2024).

Species of the genus *Bacillus* have been intensively investigated as promising biocontrol agents due to their high growth rate, strong bioactive compounds production potential, their recognition as GRAS agent (Generally Recognized as Safe) in addition to their good resistance under different environmental conditions due to their endospores forming capacity (Veras et al., 2023; Nguyen et al., 2024). *Bacillus thuringiensis* (Bt), as a *Bacillus* species, is well recognized for its ability to produce a broad spectrum of antifungal metabolites and hydrolytic enzymes such as chitinases, glucanases, and proteases, which collectively damage fungal cell walls and membranes (Osman et al., 2015; Riseh et al., 2024). Hence, Bt-based products are indeed effectively applied in agriculture field for safe and eco-friendly pest management goal (Vignesh et al., 2024).

The ability of different microorganisms and their enzymes to detoxify mycotoxins via biotransformation pathways has been well identified. Such process was reported under mild conditions by converting mycotoxins into non-toxic or less toxic compounds. There are various methods by which mycotoxins can be detoxified by microbes, such as hydrolysis, de-epoxidation, acetylation, oxidation, ring or side-chain cleavage, and glycosylation (Okoye et al., 2025). Other researchers have addressed various approaches to increasing microbial detoxification activity, such as random mutagenesis and fermentation optimization (Liu et al., 2024). This study explores the use of sub-lethal gamma irradiation as a specific biostimulant agent, based on existing physical methods in biotechnology. Rather than aiming for microbial inactivation, this approach explores the deliberate potentiation of a biological control agent’s metabolites to enhance their practical efficacy. The primary objective is to evaluate the optimization of enzymatic activity for mycotoxin degradation, supported by *in silico* molecular insights, to provide a sustainable solution for food safety. While the concept of radiation-induced bio-stimulation has been noted (Rostami et al., 2024), its specific application to augment OTA detoxification by *B. thuringiensis*, supported by detailed molecular and computational evidence, remains a novel contribution to the field of food safety and biocontrol.

Therefore, the objectives of this study are to isolate and identify a native ochratoxin A (OTA)-producing *A. niger* strain from local Egyptian wheat grains and confirm its toxigenic potential. On the other hand, a native *B. thuringiensis* isolate was evaluated for its intrinsic antifungal activity against this fungal isolate with testing its hydrolytic enzymes’ productivity. Furthermore, the potential of low-dose gamma radiation as a new bioprocessing tool in enhancing the bioactivity of the *B. thuringiensis* cell-free filtrate and the optimal irradiation dose for enhancement was determined. Subsequently, the objectives included assessing and comparing the efficacy of non-irradiated versus optimally irradiated filtrates in inhibiting fungal growth and degrading OTA into the less toxic ochratoxin α (OTα), both in liquid culture and on contaminated wheat grains. Finally, in a trial to elucidate and clarify the underlying mechanisms that are responsible for the enhanced activity, radiation-induced changes in the filtrate constituents have been monitored using a combination of enzymatic assays and Fourier transform infrared spectroscopy (FTIR) and supported by molecular docking *in silico* interpretations.

## Materials and methods

### Isolation, purification, and microscopic characterization of *Aspergillus niger* from wheat grains

Wheat grain samples were aseptically collected from local Egyptian stores and transported to the laboratory for processing within 24 h to isolate *A. niger*. Potato Dextrose Agar (PDA; HiMedia, India) fortified with 0.005% (w/v) Rose Bengal was utilized to inhibit the bacterial colonies and allow fungal growth. Ten grams of wheat grains were subjected to surface sterilization with 1% sodium hypochlorite (NaOCl) for 2 min to eliminate any external contaminants. The sodium hypochlorite was then eliminated by extensive rinsing in sterile distilled water three times. The sterilized whole grains were then placed directly on PDA plates with 0.005% Rose Bengal, with 6 grains per plate so that the fungi would be able to grow on the plate. Plates were incubated at 25 °C and kept in dark conditions in order to stimulate fungal growth. Purification was done on colonies that had the typical morphology of black spores of *A. niger*. Purification was done by subculturing fragments of *A. niger* colonies at the periphery onto fresh PDA plates containing 0.005% Rose Bengal, and incubated under the same conditions. To be examined under a microscope, purified colonies were placed on slides and examined under a compound microscope using a 40× magnifying lens to identify *A. niger* morphology, paying attention to conidiophores and conidial head structure (Jayaram and Nagao, 2018).

### Detection of ochratoxin A biosynthetic gene in *Aspergillus niger* isolate

*Aspergillus niger* isolate was cultured on Potato Dextrose Agar at 25 °C for 7 days to obtain fungal biomass. Genomic DNA was extracted from 100 mg of mycelium using the DNeasy Plant Mini Kit (Qiagen, Germany), and its quality was assessed with a NanoDrop 2000 Spectrophotometer. To evaluate the specific toxigenic potential of the *A. niger* isolate, PCR amplification was performed targeting the ochratoxin A biosynthesis gene cluster, the polyketide synthase (*pks*) gene, using target-specific primers (forward: CTTCCTTAGGGGTGGCACAGC; reverse: GTTGCTTTTCAGCGTCGGCC) (Gull Naz et al., 2023). The presence of a 400 bp amplicon confirmed the genetic capacity of the isolate to produce OTA. Following molecular identification, the toxigenic potential of the isolate was subsequently confirmed by direct detection of OTA production via HPLC analysis.

### Evaluation of antifungal activity of soil isolated bacteria against *Aspergillus niger* using dual-culture technique

The antifungal activity of bacteria, previously isolated from soil at Helwan University, Egypt, against *A. niger* was evaluated using the dual-culture technique. *Aspergillus niger*, maintained on Potato Dextrose Agar (PDA, HiMedia, India) at 25 °C, was used in the assay where a 5 mm fungal disk from a 7-day-old culture was placed 40 mm from the center of a PDA plate, opposite to a 10 mm diameter ring of the bacterial isolate, inoculated 40 mm away. Plates were incubated at 25 °C for 7 days in triplicate, with control plates containing only the *A. niger* disk. The radial growth of *A. niger* was measured, and the inhibition percentage was calculated as [(C - T) / C] × 100, where C is the radial growth diameter in the control plate (mm) and T is the radial growth diameter in the dual-culture plate (mm) (Ramona, 2021).

### Molecular identification of the isolated bacteria and fungal isolate

Molecular identification of bacterial and fungal isolates from soil and wheat grains was performed using PCR targeting universal ribosomal regions. Bacterial DNA was extracted from cultures grown in nutrient broth (HiMedia, India) at 37 °C for 48 h with 150 rpm shaking, using the DNeasy Blood & Tissue Kit (Qiagen, Germany). Fungal DNA was extracted from cultures grown on PDA (HiMedia, India) at 25 °C for 5 days, using the Fungal DNA Extraction Kit (Zymo Research, United States). The bacterial 16S rRNA gene was amplified with primers 27F (5′-AGAGTTTGATCCTGGCTCAG-3′) and 1492R (5′-GGTTACCTTGTTACGACTT-3′), and the fungal 28S rRNA gene was amplified with primers LR0R (5′-ACCCGCTGAACTTAAGC-3′) and LR5 (5′-TCCTGAGGGAAACTTCG-3′). PCR reactions (50 μL) contained 25 μL 2X Taq PCR Master Mix (Thermo Fisher Scientific, United States), 1 μL of each primer (10 μM, Integrated DNA Technologies, United States), 2 μL template DNA (50 ng/μL), and nuclease-free water (Sigma-Aldrich, United States), run on a Bio-Rad T100 Thermal Cycler with initial denaturation at 94 °C for 5 min, 35 cycles of 94 °C for 30 s, 55 °C for 30 s, and 72 °C for 1 min, and a final extension at 72 °C for 10 min. PCR products were analyzed on a 1.5% agarose gel with GelRed staining, purified using the QIAquick PCR Purification Kit (Qiagen, Germany), and sequenced via Sanger sequencing (Applied Biosystems 3730xl DNA Analyzer). Sequences were compared to the NCBI database using BLAST. For the phylogenetic analysis, reference sequences were obtained from GenBank and aligned with our isolate sequences using the ClustalW algorithm. Neighbor-joining phylogenetic trees were built using MEGA X. Evolutionary distances were calculated using the Kimura 2-parameter method, and the robustness of the branches was determined by bootstrap testing (1,000 replicates) (Sa’diyah et al., 2021; Badawy et al., 2022).

### Comparative *in silico* genomics with a reference *Bacillus thuringiensis* genome

The publicly available genome of *Bacillus thuringiensis* IBL 4222 served as a reference framework to conduct a comparative *in silico* genomic study aiming to explain the genetic characteristics that are generally attributable to the biocontrol activity of this species. All analyses were based on the whole genome sequence of *B. thuringiensis* IBL 4222, which was obtained from the NCBI GenBank database (accession number CM000759.1) to be used as the base blueprint for all further analyses. An extensive taxonomic and phylogenomic characterization of the reference strain was subsequently done with the Type (Strain) Genome Server (TYGS^1^), which provides a more robust, whole-genome-based context at much higher resolution and accuracy than single-gene methods (Lagesen et al., 2007; Camacho et al., 2009; Meier-Kolthoff et al., 2013, 2022; Lefort et al., 2015; Ondov et al., 2016; Kreft et al., 2017; Meier-Kolthoff and Göker, 2019). Data on the inferred functional potential of this biocontrol agent was obtained by annotating its genome with the RAST (Rapid Annotation using Subsystems Technology^2^) service; the service identified the presence of conserved protein-encoding genes and metabolic subsystems, thus revealing the common genetic machinery behind the antifungal and detoxifying properties typical of *B. thuringiensis* (Brettin et al., 2015). Lastly, the overall genome architecture and gene organization could be visualized using a circular map created with the Proksee web tool (version 1.0.0, developed by the Stothard Research Group and the National Microbiology Laboratory of Canada^3^), which provided information about the structural and compositional characteristics of the genome (Grant et al., 2023).

### Plate assays to measure the production of chitinase, glucanase, and protease by *Bacillus thuringiensis*

A specific plate assay was done to evaluate the production of chitinases, glucanases, and proteases by the *B. thuringiensis* strain through direct bacterial inoculation. The nutrient broth (HiMedia, India) was inoculated with bacterial cultures and incubated at 37 °C with a shaking rate of 150 rpm. In the case of the chitinase assay, the cultures were inoculated on chitin agar plates (including 125 mL colloidal chitin, 0.65 g Na_2_HPO_4_·2H_2_O, 1.5 g KH_2_PO_4_, and 12.25 g NaCl) (Asif et al., 2020). In the case of the glucanase assay, cultures were inoculated on glucan agar plates (0.2% glucan, 0.1% (NH_4_)2SO_4_, 0.05% MgSO_4_·7H2O, 0.1% KH_2_PO_4_, and 1.5% agar) (Mufida et al., 2024). To perform the protease assay, cultures were incubated on gelatin agar plates (5.0 g NaCl, 10.0 g peptone, 10.0 g beef extract, 18.0 g agar, and 4.0 g gelatin per liter) and incubated at 37 °C for 72 h; protease activity was determined by applying mercuric chloride reagent. This particular reagent was chosen because of its capacity to result in a very clear visual precipitate of unhydrolyzed gelatin in qualitative screening. All the heavy metal waste generated was strictly managed and disposed of under institutional hazardous waste procedures. Gelatin hydrolysis was detected by the use of the reagent showing distinct halos (Mohamed et al., 2023).

### Antifungal activity of non-irradiated and gamma-irradiated cell-free filtrates of *Bacillus thuringiensis* against *Aspergillus niger*

A cell-free filtrate assay was used to determine the antifungal action of non-irradiated and gamma-irradiated cell-free filtrates of *B. thuringiensis* strain CU-BT1 against *A. niger* strain CU-AN1. To begin with, *B. thuringiensis* was grown in nutrient broth (HiMedia, India) at 37 °C with a shake speed of 150 rpm for 24 h of incubation. The culture was then centrifuged, and the supernatant was filtered using a 0.22 μm syringe filter to ensure the filtrate was completely sterile and free from microbial contamination. This filtrate was further separated into two sets. One of them was the non-irradiated control, and the other one was gamma-irradiated at doses of 0.1 to 20 kGy using a Co-60 gamma irradiator (Gamma Chamber 4000 A, NCRRT, Cairo, Egypt) with an average dose rate of 0.624 kGy/h. The corresponding irradiation times were 9.6 min (0.1 kGy), 19.2 min (0.2 kGy), 48.1 min (0.5 kGy), 96.2 min (1 kGy), 8.0 h (5 kGy), 16.0 h (10 kGy), 24.0 h (15 kGy), and 32.1 h (20 kGy). Irradiation was done strictly under temperature-controlled conditions, with the samples kept at around 4 to 8 °C in a chilled environment to isolate radiolytic effects from thermal degradation. In order to ensure a robust control baseline, the non-irradiated samples were also held at these same cold temperatures (4–8 °C) alongside the irradiated samples for the same period as the longest irradiation exposure time. Once treated, all samples (irradiated and non-irradiated) were immediately aliquoted and frozen at −20 °C to prevent further radiolytic or autoproteolytic reactions, thus fully preserving the enzymes for downstream analysis. Chilled conditions were also necessary to prevent the autoproteolytic degradation of enzymes by endogenous proteases during the relatively long exposure times. Moreover, this extensive dose range was chosen particularly to trace the entire biphasic dose–response curve. The limit was purposely set to sterilization doses (20 kGy) to clearly define the maximum dose of enzyme radiotolerance and to verify the threshold of total structural inactivation, thus justifying the specific hormetic bio-stimulation found at sub-lethal doses. The poisoned agar technique was used to test the antifungal activity. Briefly, the cell-free filtrate (non-irradiated or irradiated) was added to molten PDA (cooled to around 45 °C) just before the plates were poured, resulting in a final concentration of 100 μL/mL (10% v/v) (Tang et al., 2026). This particular level was chosen to offer a robust screening dose without affecting the structural gelling integrity of the agar. After the amended plates had solidified, a 5 mm fungal disk of *A. niger* (grown on PDA at 25 °C) was inoculated at the center of each plate. The plates were then incubated at 25 °C for 7 days in triplicate, with control plates being prepared by adding the same volume of sterile nutrient broth in lieu of the filtrate. *A. niger* radial growth was measured, and the percentage of inhibition was calculated as [(C - T) / C] × 100, where C is the control radial growth diameter (mm) and T is the radial growth diameter of the treated plates (mm) (Yassein and Elamary, 2021).

### Evaluation of ochratoxin A biodegradation and ochratoxin α production by non-irradiated and gamma-irradiated cell-free filtrates of *Bacillus thuringiensis* in liquid culture and on contaminated wheat grains

The biodegradation of OTA and production of OTα by non-irradiated and gamma-irradiated (0.2 kGy) cell-free filtrates of *B. thuringiensis* were evaluated in two experiments. In the first, 100 μL of each filtrate was incubated with 1 mL of *A. niger* spores (1 × 10^3^ spores/mL) in yeast extract sucrose (YES) broth (20 g yeast extract, 40 g sucrose per 1,000 mL distilled water) at 25 °C for 7 days. OTA and OTα were extracted using HPLC-grade chloroform (Sigma-Aldrich, United States), separated via a funnel, evaporated with a Stuart RE300 Rotary Evaporator (Cole-Parmer, UK), and reconstituted in 1 mL acetonitrile. The concentrations were quantified using a Shimadzu Prominence HPLC system (Shimadzu, Japan) equipped with a fluorescence detector (FLD). The precision of the target mycotoxin quantification is a consequence of the inherent optical selectivity of the detection system. Using a mobile phase comprising acetonitrile:water:acetic acid (49:49:2, v/v/v) at a flow rate of 1 mL/min, the target mycotoxins were clearly resolved, with peaks at 10.5 and 4.8 min for OTA and OTα, respectively. In the second experiment, 30 g of autoclaved wheat grains, inoculated with 1 mL of *A. niger* spores (1 × 10^3^ spores/mL) and incubated at 25 °C for 5 days to induce OTA production, were treated with 50 mL of each filtrate and incubated for an additional 7 days. The grains were ground into powder using a Waring Commercial Blender, and OTA and OTα were extracted with 50 mL chloroform, followed by HPLC analysis as described. In both experiments, OTA reduction percentages were calculated as [(C - T) / C] × 100, where C is the control OTA concentration (μg/mL for broth, μg/kg for grains) and T is the treated OTA concentration (Yassein and Elamary, 2021).

### Fourier transform infrared spectroscopy spectral analysis of structural changes in gamma-irradiated *Bacillus thuringiensis* cell-free filtrate

Fourier transform infrared spectroscopy for detection of structural changes of non-irradiated and irradiated cell-free filtrates of *B. thuringiensis* was performed on a BRUKER Tensor 27 spectrometer with an ATR accessory (ZnSe crystal) scanning 400 to 4,000 cm^−1^ at 4 cm^−1^ resolution with 32 scans using 99.9% ethanol (Merck) to clean the crystal between samples. To ensure strict comparability, these measurements were conducted on aliquots from the same homogenous batch of non-irradiated and irradiated filtrates, which had been subjected to the identical temperature-controlled conditions (4–8 °C) and equivalent holding times prior to freezing at −20 °C (Stanca et al., 2023).

### Determination of carboxypeptidase enzyme activity in *Bacillus thuringiensis* cell-free filtrate

The enzymatic activity of carboxypeptidase in cell-free filtrates from non-irradiated and gamma-irradiated (0.2 kGy) *B. thuringiensis* cultures was evaluated using a spectrophotometric assay with N-acetyl-L-phenylalanyl-3,5-diiodo-L-tyrosine (Sigma-Aldrich) as the substrate. The assay involved mixing 0.1 mL of filtrate with 2.9 mL of 0.1 M Tris–HCl buffer (pH 7.5) containing 1 mM substrate, followed by incubation at 37 °C for 30 min. The change in absorbance, indicative of enzymatic activity, was measured at 410 nm using a Shimadzu UV-1800 UV–Vis Spectrophotometer. Enzyme activity was estimated by a pre-established carboxypeptidase enzyme standard curve obtained for standard carboxypeptidase enzyme (Sigma-Aldrich) at concentrations of 0.1, 0.3, 0.5, 0.7, 0.9, and 1.0 U/mL (Calicis et al., 2025).

### *In silico Bacillus thuringiensis* carboxypeptidase and radiation-enhanced carboxypeptidase docking with ochratoxin A

This study focused on the structure optimization and docking of carboxypeptidase from *B. thuringiensis*, using the AlphaFold-predicted model of a representative sequence (UniProt ID: A0A437SN38) loaded into the Schrödinger Maestro 13.4 software. Since there was no strain-specific sequence information for the native isolate, this well-studied sequence was used as a model archetype. Since the underlying active site structures, substrate-binding modes, and core scaffolds of these hydrolytic enzymes are remarkably similar in *Bacillus*, this model is a valid and reliable 3D representation to theoretically assess the basic binding modes with the substrate and the localized effects of radiation-induced modifications (Rimsa et al., 2014; Minato et al., 2020; Chandravanshi et al., 2024). The wild-type structure was pre-processed using the Protein Preparation Wizard, which added hydrogens, refined the hydrogen bond network, and carried out restrained energy minimization with the OPLS4 force field until the heavy-atom root mean square deviation (RMSD) was 0.30 Å (Calicis et al., 2025). A theoretical *in silico* model of the potential structural effects of radiation-induced oxidative stress on carboxypeptidase activity was built using the Mutate Residues tool in Maestro. Although gamma radiation causes non-specific and extensive oxidative modifications in the secretome, representative modifications were modeled to explore the impacts of potential changes in electrostatics and oxidation on binding. The basis for the choice of these residues is that Met140 and Tyr198 are highly solvent-exposed and, as such, are highly vulnerable to radiolytic reactive oxygen species (ROS), and are also in relatively close proximity to the active site where modifications can affect substrate binding. The specific modifications were the conversion of methionine 140 to methionine sulfoxide (MSO) (Radomska and Wolszczak, 2023) and tyrosine 198 to hydroxylated tyrosine (TYR-OH) (Demasi et al., 2021). In addition, a hypothetical charge-inversion modification (aspartic acid 142 to arginine) was introduced. We explicitly state that without experimental confirmation from mass spectrometry, these specific modifications are not absolute mappings, but can be used as a rigorous theoretical proof-of-concept substitute to illustrate how radiation-induced local structural and electrostatic changes can mechanistically improve the binding interactions between an enzyme and its substrate (Davies, 2016). Ochratoxin A (PubChem CID: 442530) was prepared with the LigPrep tool (Schrödinger, LLC, New York, NY) to generate tautomers and ionization states (pH 7.5 ± 2.0) and minimize energy. Docking simulations were carried out using Glide (Extra Precision - XP) with OTA as the ligand for the native and irradiated carboxypeptidase structures. Structural changes due to irradiation were evaluated by calculating the instability index, hydrophobicity, and secondary structure using BioPython’s ProtParam (Rustagi et al., 2023).

### Statistical analysis

Unless otherwise indicated, experiments were conducted in triplicate. The results are presented as the mean ± SD. One-way ANOVA and Tukey’s *post hoc* test (*p* < 0.05) were used to assess the statistical significance of differences between treatments. Data were analyzed using SPSS v.25.

### Safety considerations

Gamma irradiation was conducted at the NCRRT, Cairo, following standard operating procedures, utilizing shielding, dosimetry, and supervision.

## Results

### Molecular identification of isolated bacteria and isolated fungus

**Figure 1:**
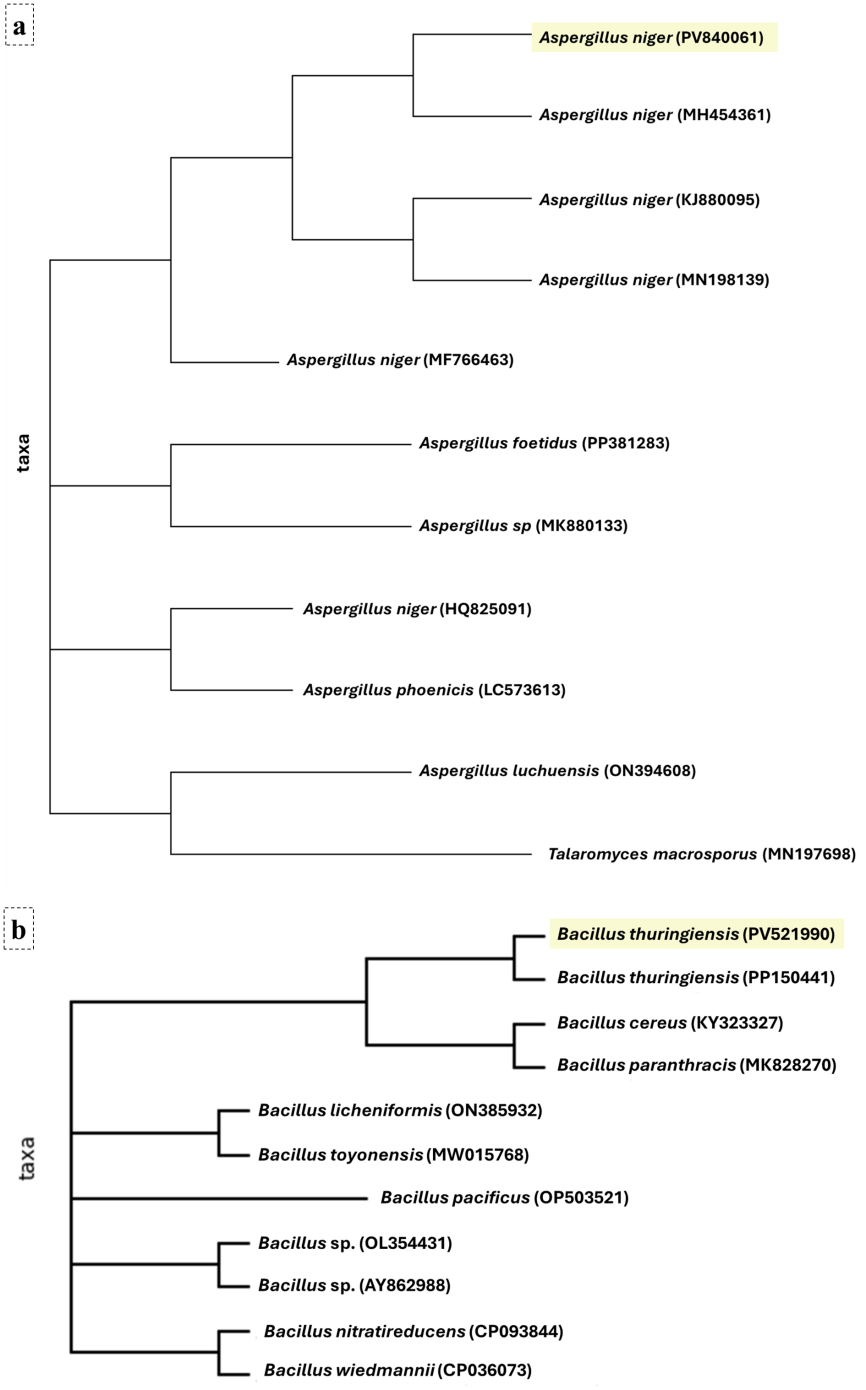
Phylogenetic analysis of fungal and bacterial isolates. **(a)** The fungal isolate groups with *Aspergillus niger* within the *Aspergillus* genus, and **(b)** the bacterial isolate clusters closely with *Bacillus thuringiensis* within the *Bacillus* genus, as depicted in the constructed phylogenetic trees.

Molecular identification assays successfully amplified the 16S rRNA gene for the bacterial isolate and the 28S rRNA gene for the fungal isolate. BLAST analysis of the bacterial sequence showed 98.11% similarity to *Bacillus thuringiensis* in the NCBI database, and the sequence was submitted to GenBank under accession number PV521990 (designated herein as laboratory strain CU-Bt1). The fungal sequence exhibited 99.0% similarity to *Aspergillus niger*, with submission to GenBank under accession number PV840061 (designated herein as laboratory strain CU-An1). Phylogenetic trees constructed confirmed these identities, with *B. thuringiensis* clustering within the *Bacillus* genus and *A. niger* within the *Aspergillus* genus, as depicted in Figure 1.

### Morphological characterization of *Aspergillus niger* isolated from wheat grains

**Figure 2:**
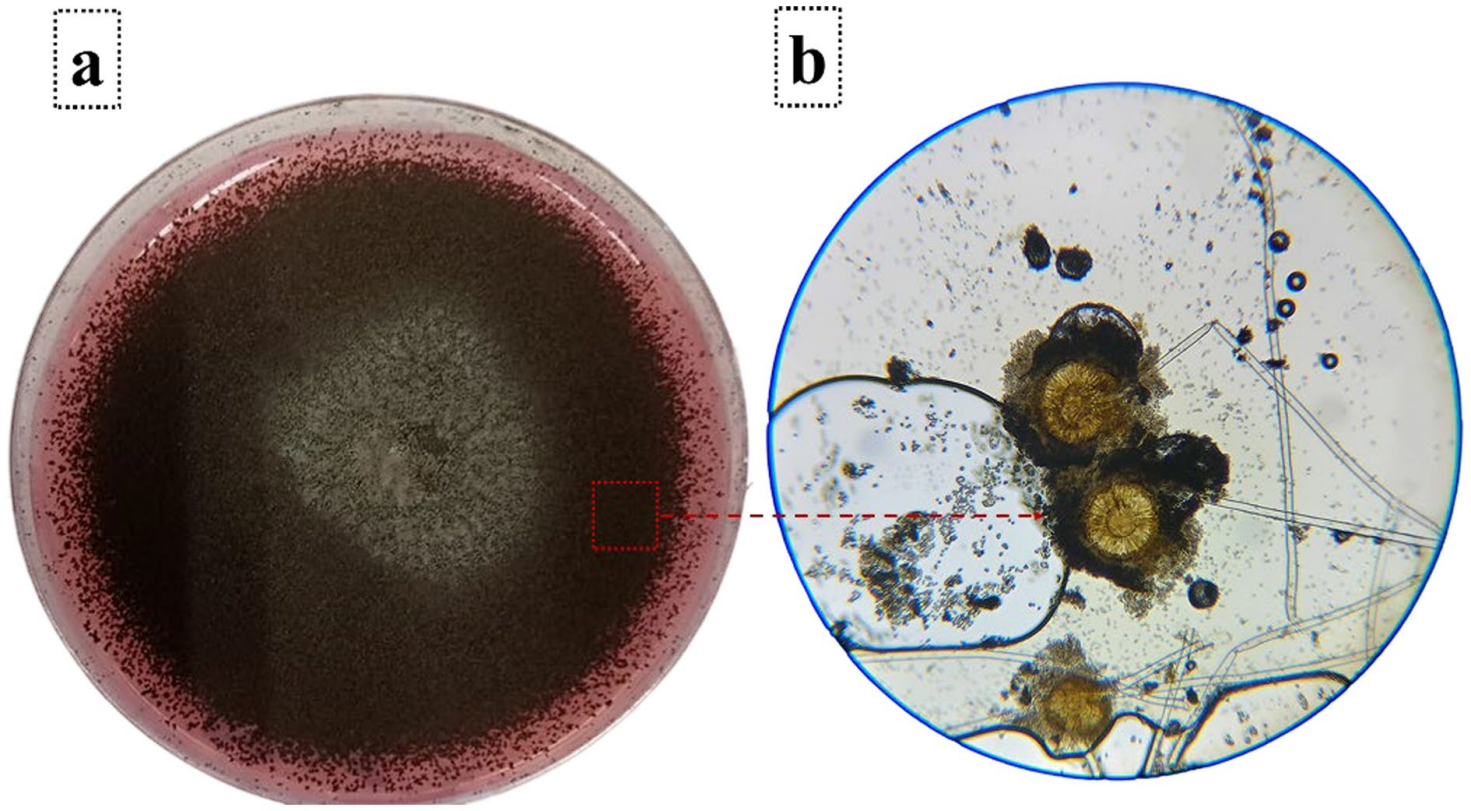
Isolated *A. niger*: **(a)** Purified *A. niger* on PDA with 0.005% Rose Bengal, displaying uniform black colonies after mycelial transfer; **(b)** *Aspergillus niger* under a light microscope showing dark-pigmented conidia at 40× magnification.

The isolation procedure resulted in viable fungal growth on the wheat grain samples. Upon incubation on PDA for 7 days, powdery blackish colonies were observed around the grains. Subsequent cultivation on fresh PDA resulted in black colonies (Figure 2a). Conidia were dark-colored, and conidiophores showed typical arrangements (Figure 2b). These macro- and micro-morphological characteristics are in full agreement with *A. niger* morphology, which provides phenotypic confirmation of the molecular identification of strain CU-AN1 as mentioned above.

### Detection of ochratoxin A biosynthetic gene in *Aspergillus niger* isolate

**Figure 3:**
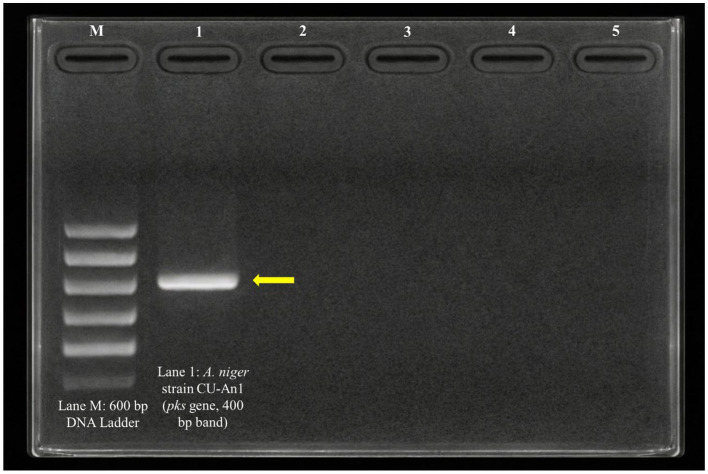
Gel electrophoresis photograph showing detection of the polyketide synthase (*pks*) gene in *A. niger*. Lane M: 600 bp DNA ladder; Lane 1: *A. niger* strain CU-An1 isolate with a 400 bp band indicating the *pks* gene.

A distinct band at 400 bp was observed on the agarose gel (Lane 1) for the *A. niger* strain CU-An1 isolate, corresponding to the expected size of the targeted OTA biosynthesis polyketide synthase (*pks*) gene (Figure 3).

### Evaluation of antifungal activity of soil isolated bacteria against *Aspergillus niger* using dual-culture technique

**Figure 4:**
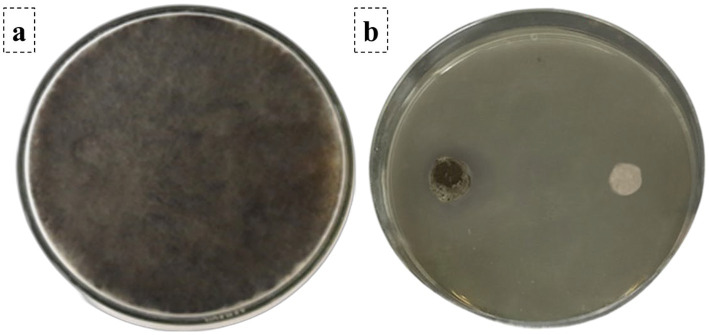
Evaluation of antifungal activity. **(a)** Control plate, *A. niger* exhibited full growth after 7 days. **(b)** Dual-culture assay demonstrating inhibition of *A. niger* by soil isolated bacteria.

The dual-culture assay demonstrated significant antifungal activity of the previously isolated soil bacteria from Helwan University against *A. niger*. In the control plates, *A. niger* exhibited 90.00 mm radial growth after 7 days. In the dual-culture plates, the radial growth of *A. niger* toward the bacterial isolate was substantially reduced, with a mean diameter of 11.47 ± 0.30 mm and the inhibition percentage was 87.26% (Figure 4).

### Genomics-driven characterization of *Bacillus thuringiensis* IBL 4222: predicting the theoretical genetic machinery for biocatalysis and detoxification

**Figure 5:**
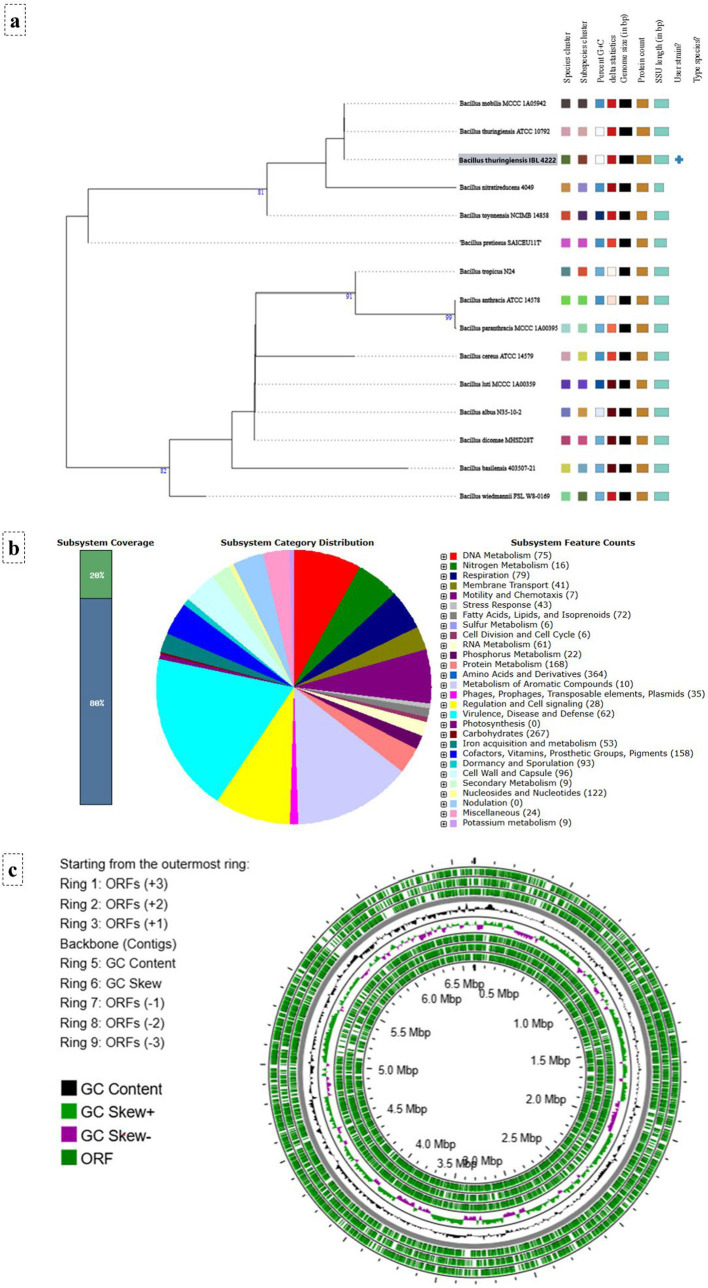
*In silico* genomic characterization of the reference strain *B. thuringiensis* IBL 4222. **(a)** Phylogenomic tree based on TYGS analysis. **(b)** Functional subsystem annotation of the genome in the RAST SEED viewer. **(c)** Circular map of the genome showing open reading frames (ORFs), GC content, and GC skew.

While whole-genome sequencing of the native isolate was precluded by resource constraints, a canonical reference genome was utilized to map the highly conserved genetic blueprints typical of this species. The *in silico* analysis began with the successful retrieval of the complete, circular chromosome of *B. thuringiensis* IBL 4222 (GenBank ID: CM000759.1), which comprised 6,612,432 bp with a G + C content of 34.8%. Whole-genome phylogenetic analysis of the reference genome using the TYGS platform robustly confirmed the reference strain’s taxonomic identity, placing it in a well-supported clade with the *B. thuringiensis* type strain with 99% GBDP branch support (Figure 5a). Functional annotation of the genome via the RAST service identified a gene-rich architecture, revealing 7,170 coding sequences and 101 RNAs distributed across 338 functional subsystems (Figure 5b). Importantly, these subsystems comprise genes that are essential to the biocontrol function predicted for our isolate, including sporulation, stress adaptation, and hydrolytic enzyme production. Lastly, the complete genome was depicted with Proksee in a circular map (Figure 5c), showing a compact genome with a symmetrical distribution of open reading frames (ORFs) on both strands and the presence of GC skew, which confirmed the high quality of the genome assembly, as well as provided information on the overall genomic structure.

### Assessment of chitinase, glucanase, and protease production by *Bacillus thuringiensis* using plate assays

**Figure 6:**
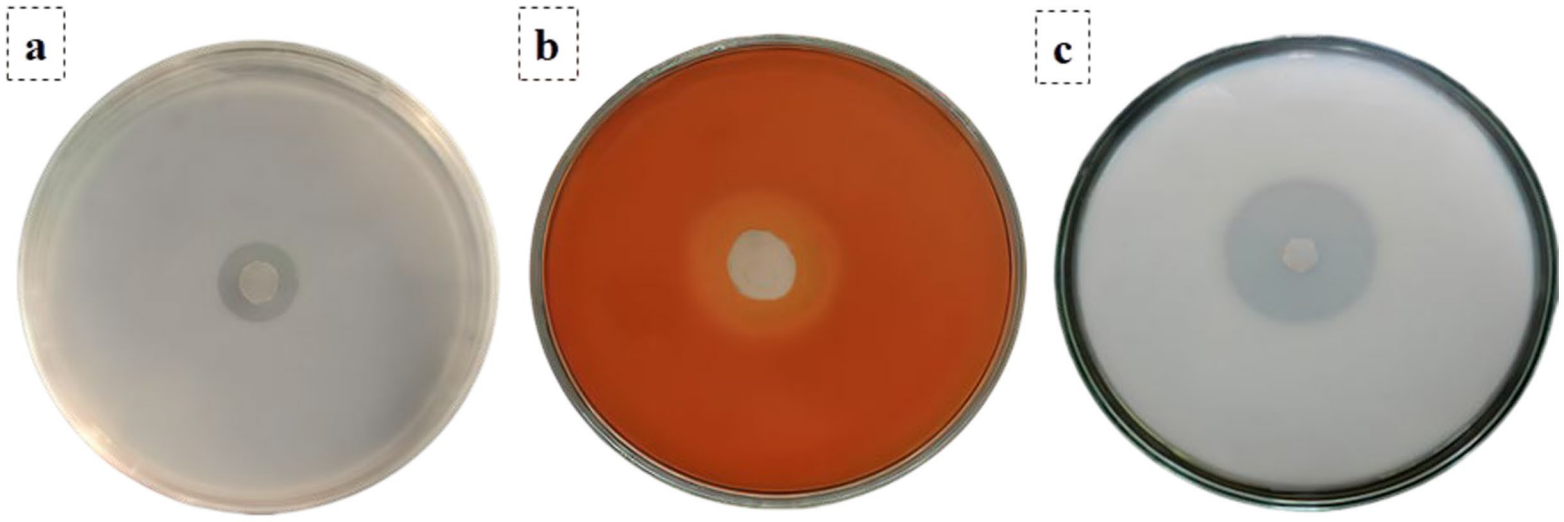
Plate assays for **(a)** chitinase, **(b)** glucanase, and **(c)** protease production by *B. thuringiensis* strain.

To elucidate the biochemical mechanisms driving the observed structural degradation of the *A. niger* cell wall, the native bacterial isolate was screened for major fungal cell-wall degrading enzymes. The plate assays confirmed the production of chitinases, glucanases, and proteases by *B. thuringiensis* strain (Figure 6). For the chitinase assay, a clear zone was around the inoculum after incubation, indicating chitinase activity through the hydrolysis of colloidal chitin. In the glucanase assay, a clear zone was present around circular inoculum after Congo Red staining, confirming glucanase activity. For the protease assay, a clear zone was detected due to gelatin hydrolysis, reflecting protease activity.

### Assessment of antifungal activity of non-irradiated and gamma-irradiated cell-free filtrates of *Bacillus thuringiensis* against *Aspergillus niger*

**Figure 7:**
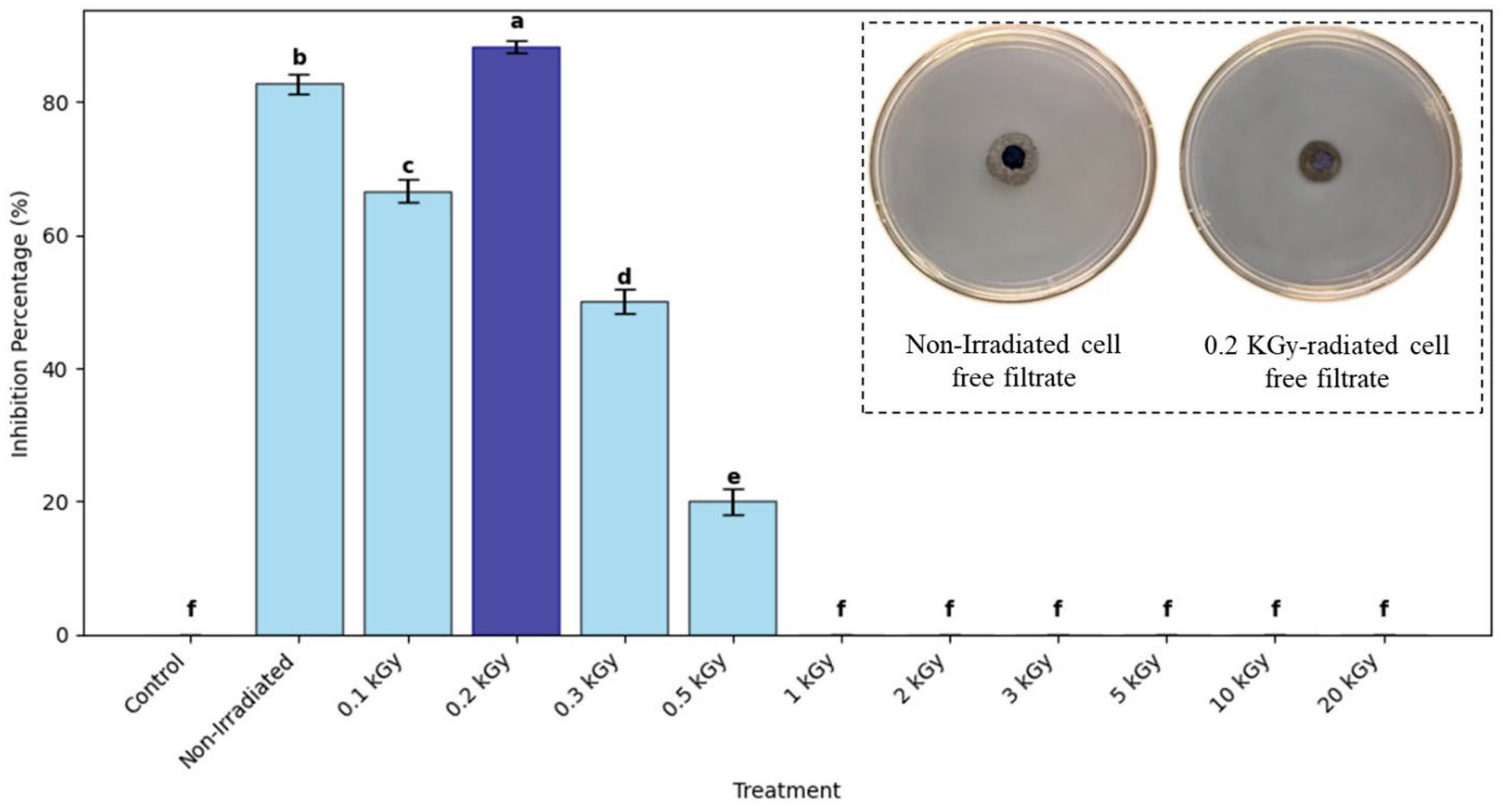
Inhibition percentage of *A. niger* by *B. thuringiensis* cell-free filtrates. Bars with different letters (a–f) indicate statistically significant differences (*p* < 0.05).

The control group (untreated *A. niger* strain CU-AN1) reached a full radial mycelial growth of 90.00 mm. The addition of non-irradiated cell-free filtrate considerably suppressed the radial growth to 15.48 ± 1.20 mm (82.80% inhibition). Interestingly, the 0.2 kGy irradiated cell-free filtrate demonstrated an increased capacity for suppression to 10.52 ± 1.00 mm (88.3% inhibition). Deviations from the optimal 0.2 kGy dose produced a significant reduction in antifungal activity across the different radiation doses. Maximum antifungal efficacy was achieved at the 0.2 kGy dose, with decreased activity accompanying increasing variations in dose, particularly showing a sharp decline at higher dosages and a complete loss of activity at doses of 1 kGy and higher, reflecting the severe structural instability and denaturation of the antifungal effector biomolecules (enzymes and lipopeptides) in response to excessive radiolytic damage (Figure 7).

### *In vitro* evaluation of OTA biodegradation and OTα production by non-irradiated and gamma-irradiated cell-free filtrates of *Bacillus thuringiensis* in liquid culture and on contaminated wheat grains

**Figure 8:**
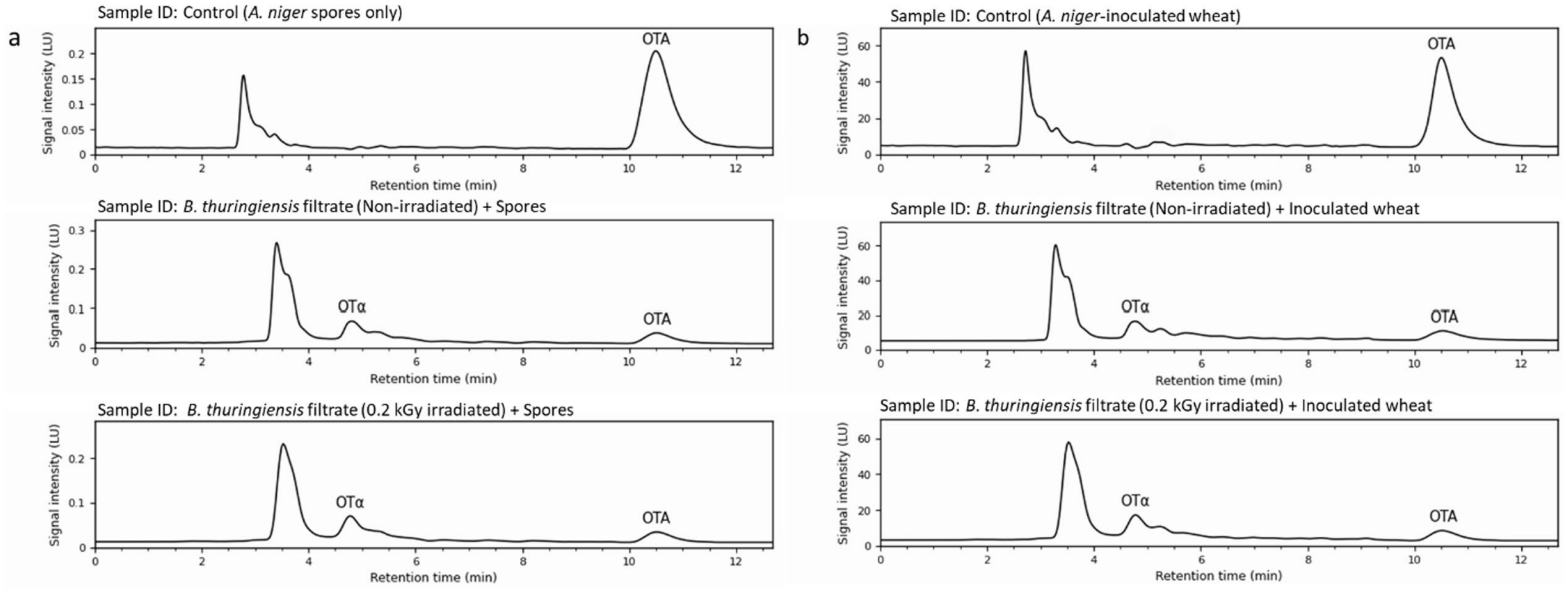
HPLC analysis of OTA degradation and OTα production by non-irradiated and gamma-irradiated (0.2 kGy) cell-free filtrates of *B. thuringiensis*: **(a)** Against *A. niger* spores in broth medium and **(b)** on contaminated wheat grains. The targeted peaks are explicitly annotated, with the degradation product Ochratoxin α (OTα) detected at a retention time of 4.8 min, and the parent toxin Ochratoxin A (OTA) detected at 10.5 min.

The biodegradation assay results for OTA and OTα production by non-irradiated and gamma-irradiated (0.2 kGy) cell-free filtrates of *B. thuringiensis* are summarized in Figure 8. In the liquid yeast extract sucrose (YES) broth assay (Figure 8a), the control with *A. niger* spores showed an OTA concentration of 0.059 ± 0.002 μg/mL. The 0.2 kGy irradiated biocatalytic filtrate demonstrated a superior targeted bioconversion, reducing OTA to 0.008 ± 0.001 μg/mL (86.78 ± 0.60% reduction) with a concurrent increased production of OTα (0.051 μg/mL). Crucially, this enhanced biotransformation rate directly correlates with the 18.06% increase in carboxypeptidase catalytic activity observed in the spectrophotometric assays, strongly suggesting a targeted enzymatic bioprocess driven by this class of enzymes. In the wheat grain assay (Figure 8b), the control with *A. niger*-inoculated wheat grains exhibited an OTA concentration of 14.470 ± 0.150 μg/kg. Non-irradiated filtrate reduced OTA to 3.355 ± 0.090 μg/kg (76.81 ± 0.62% reduction) with a quantified OTα production of 11.115 μg/kg, while the 0.2 kGy irradiated filtrate reduced OTA to 2.878 ± 0.080 μg/kg (80.12 ± 0.55% reduction) with a quantified OTα production of 11.592 μg/kg. Although the percentage point increases were modest, one-way ANOVA confirmed that the improvements in OTA reduction by the irradiated filtrate were statistically significant (*p* < 0.05) in both the liquid broth and wheat grain assays.

### FTIR spectral analysis of structural changes in gamma-irradiated *Bacillus thuringiensis* cell-free filtrate and its effects on antifungal activity and ochratoxin degradation

**Figure 9:**
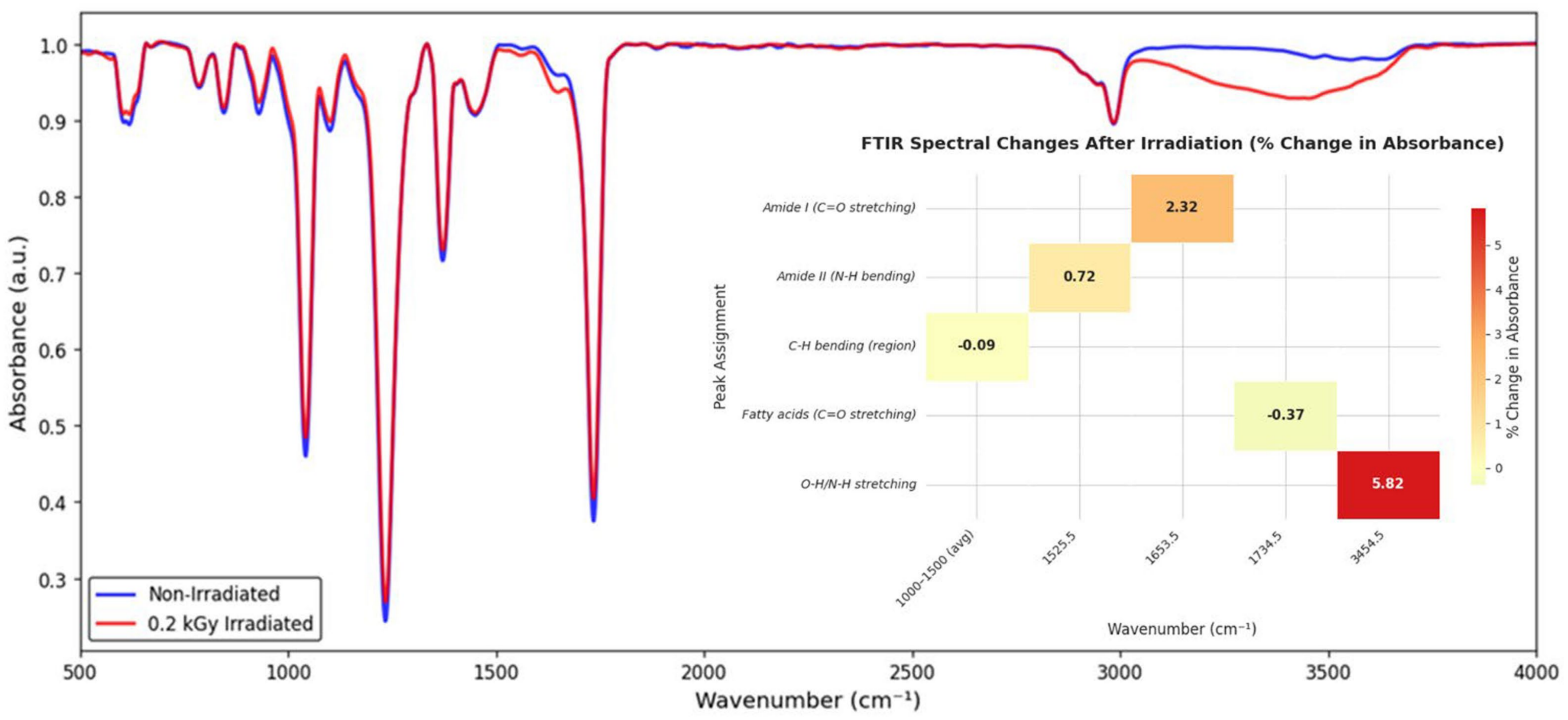
FTIR spectral comparison of non-irradiated and 0.2 kGy irradiated cell-free filtrates of *B. thuringiensis* strain CU-BT1, highlighting radiation-induced structural modifications and the corresponding percentage changes in absorbance.

FTIR spectral analysis of the *B. thuringiensis* cell-free filtrate revealed notable structural changes post-irradiation at 0.2 kGy (Figure 9). Absorbance at 1653.5 cm^−1^ (Amide I, C=O stretching) increased from 0.93712 to 0.95889 (+2.32%), suggesting enhanced protein interactions, while at 1525.5 cm^−1^ (Amide II, N-H bending), it rose from 0.99132 to 0.99843 (+0.72%), indicating subtle secondary structure shifts. The absorbance in the fatty acid region at 1734.5 cm^−1^ (C=O stretching) decreased slightly from 0.91044 to 0.90710 (−0.37%), and a significant increase occurred at 3454.5 cm^−1^ (O-H/N-H stretching) from 0.92872 to 0.98277 (+5.82%), reflecting oxidative byproduct formation. An average absorbance decrease from 0.94382 to 0.94298 (−0.09%) occurred in the 1,000–1,500 cm^−1^ region (C-H bending), indicating changes in aliphatic structures.

### Determination of carboxypeptidase enzyme activity in gamma-irradiated *Bacillus thuringiensis* cell-free filtrate

**Figure 10:**
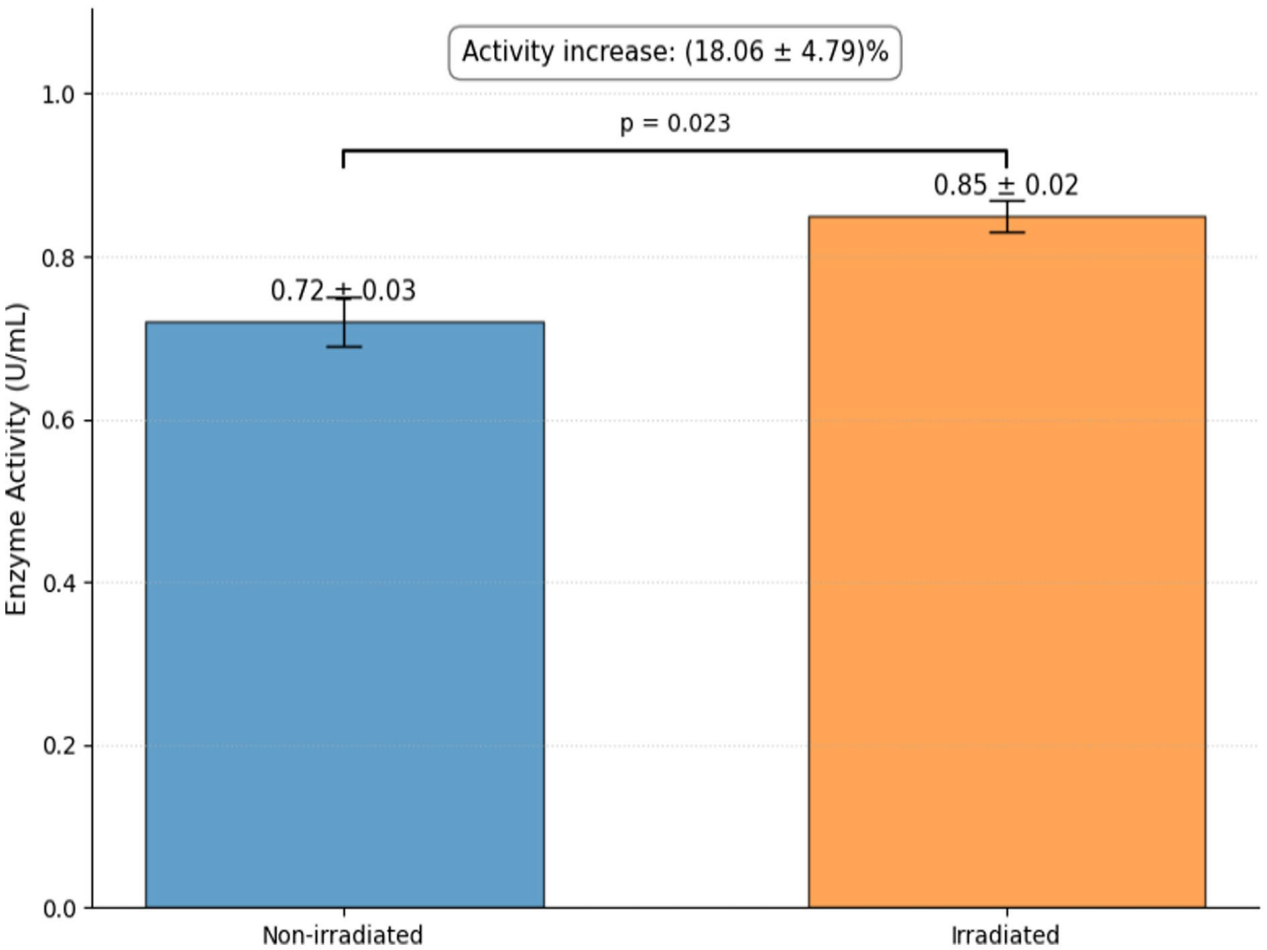
Effect of gamma irradiation on carboxypeptidase activity. Bars represent mean ± SD (*n* = 3). Irradiated filtrates showed a significant increase in enzyme activity compared to non-irradiated samples (*p* = 0.023).

The spectrophotometric results show the difference in carboxypeptidase activity between non-irradiated and 0.2 kGy irradiated *B. thuringiensis* cell-free filtrate (Figure 10). The non-irradiated and irradiated filtrates had activities of 0.72 ± 0.03 U/mL and 0.85 ± 0.02 U/mL, respectively. The gamma-irradiated sample had an 18.06% higher activity than the non-irradiated sample. Since the treatments were sourced from the exact same (fully homogenous) batch of initial secretome, the total protein concentration in the samples was the same; hence, this measured increase in volumetric activity is directly and proportionally translated into an enhancement in the specific activity of the enzyme.

### Radiation-induced enzyme optimization: *in silico* structural analysis of native and optimized carboxypeptidase interactions with ochratoxin A

**Figure 11:**
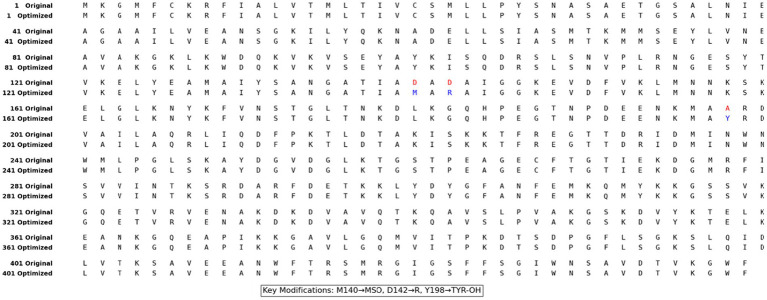
Sequence of the original (canonical) protein and the theoretical “proxy” sequence. Letters are in red (original residue) and blue (modeled proxy) to show the amino acid substitutions used to model radiation-induced local electrostatic effects in docking simulations.

**Figure 12:**
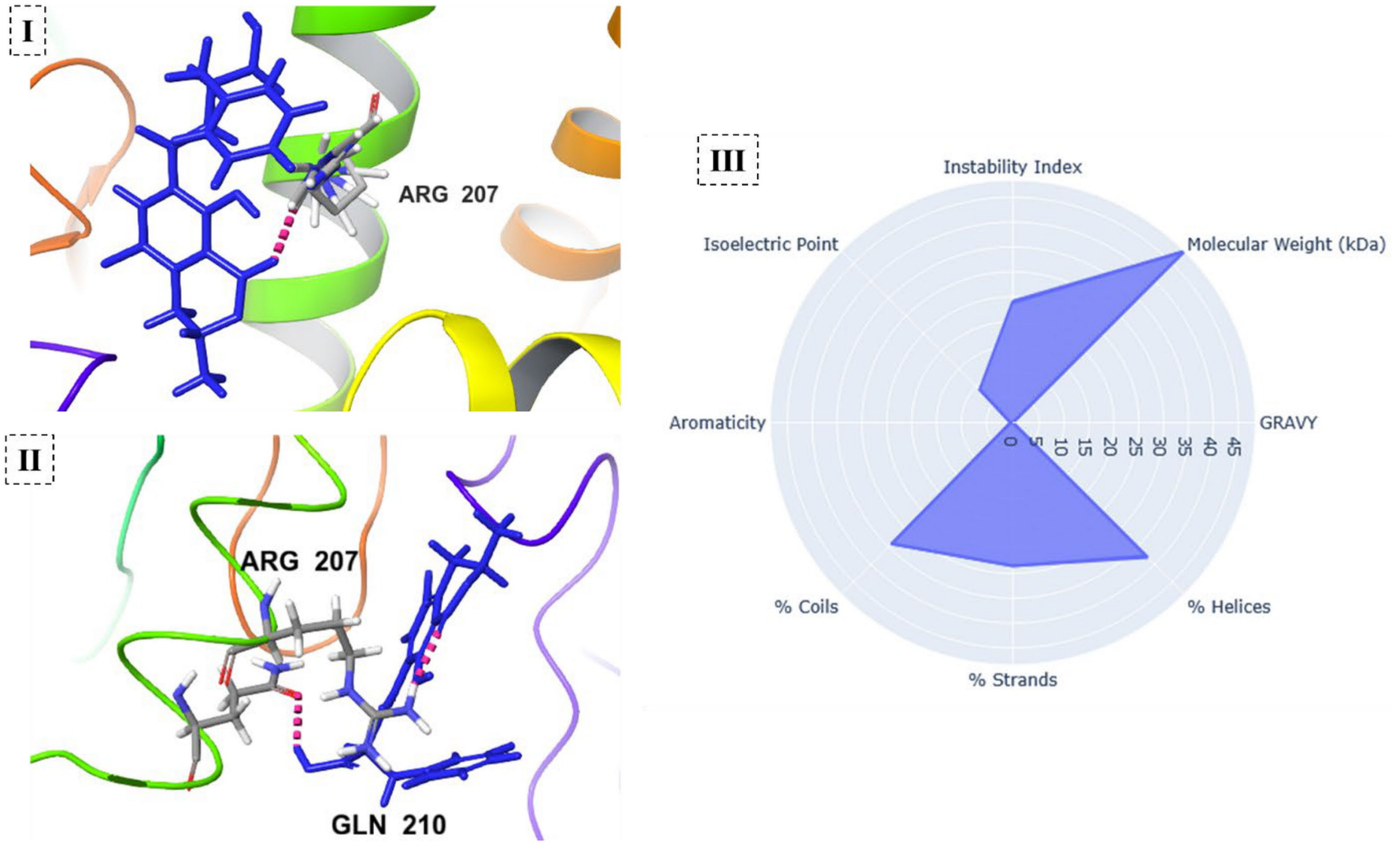
Docking interactions of native **(I)** and optimized **(II)** carboxypeptidase from *B. thuringiensis* with OTA. **(III)** Stability of native and optimized carboxypeptidase.

To provide a theoretical mechanistic framework explaining the enhanced *in vitro* enzymatic activity, a canonical representative model of *B. thuringiensis* carboxypeptidase was utilized as a structural proxy. The computational analysis revealed significant improvements in both stability and functional performance of the gamma-irradiated (optimized) carboxypeptidase. Structural stability calculations revealed a 1.6% reduction in the instability index (from 24.11 to 23.72) and increased hydrophilicity (GRAVY score from −0.354 to −0.368), indicating enhanced stability. Docking studies showed improved binding of the irradiated enzyme to OTA (GlideScore from −7.456 to −7.992 kcal/mol). This improvement was associated with the formation of a new hydrogen bond between the modified Tyr-OH198 and Gln210, in addition to the original hydrogen bond with Arg207. There were no significant differences in the secondary structure (a 1.2% decrease in α-helical content from 38.04 to 37.59%, while the β-strand content remained identical at 28.47%), suggesting that the modifications did not alter the overall conformation, but refined the active site. The isoelectric point increased from 9.05 to 9.17 because of the positive charge introduced at position 142 (Figures 11, 12).

## 4 Discussion

Ochratoxin A (OTA) contamination in agricultural products such as wheat remains a global food safety concern, and effective biotechnological interventions are needed to tackle this issue (Wang et al., 2024). Selective enzymatic biotransformation of OTA to non-toxic degradation products is an effective process. In particular, the amide hydrolysis of OTA by carboxypeptidase plays a pivotal role in bioprocessing (Xiong et al., 2020; Ábrahám et al., 2025). In this study, we examined the use of a natural soil isolate of *B. thuringiensis* strain CU-BT1 as a biocontrol agent for OTA-producing *A. niger* strain CU-AN1. Employing a native soil microorganism harnesses its natural ability to adapt and compete in its natural habitat, making it feasible for agricultural and food safety practices. This study identified the antifungal properties of *B. thuringiensis* and explored the use of low-dose gamma radiation to improve its biocontrol and detoxification potential. This work concurs with local research on the presence of *A. niger* in North African cereals, in addition to other black Aspergilli such as *Aspergillus tubingensis*, which calls for sustainable detoxification methods to combat this food safety threat (Gherbawy et al., 2025).

The toxigenic capacity of the *Aspergillus niger* isolate was confirmed by the detection of a 400 bp fragment of the OTA biosynthesis gene *pks* gene, which is a part of the OTA gene cluster, consistent with the reported biosynthetic pathway (Esuola and Ortega-Beltran, 2025). The antifungal efficacy of the *B. thuringiensis* strain, which caused an 87.26% inhibition of *A. niger* growth, is biologically supported by the results of this study that confirm the secretion of a complex cocktail of lytic enzymes. The qualitative tests indicated the isolate’s secretion of proteases, glucanases, and chitinases, whose antifungal action resulted in a cooperative and sequential degradation of the fungal cell wall (Osman et al., 2015; Riseh et al., 2024; Vignesh et al., 2024). This begins with proteases that disrupt the outer protein wall, followed by the underlying polysaccharides. Subsequently, glucanases and chitinases hydrolyze the structural β-glucan and chitin polymers, respectively, which compromises the cell wall’s integrity and leads to osmotic instability and lysis (Yuan et al., 2008; Riseh et al., 2024). The notable efficacy of this multi-enzyme activity is supported when compared to recent literature; the observed inhibition compares favorably to the 58% by *Bacillus cereus* reported by Yi et al. (2023) and the 89% by *Bacillus amyloliquefaciens* noted by Zhang et al. (2024). The significance of this enzymatic strategy is further corroborated by studies from Li et al. (2023) and Zhao et al. (2022), which link the co-production of such enzymes in *Bacillus* to potent antifungal outcomes. Therefore, the combination of superior growth inhibition and a well-defined lytic enzyme arsenal, as demonstrated by our isolate, validates its strong potential as a biocontrol agent.

While our *in vitro* biochemical and plate assays empirically confirmed the production of chitinases, glucanases, proteases, and specifically carboxypeptidase by our native isolate, the *in silico* genomic analysis and molecular docking utilized the reference strain (*B. thuringiensis* IBL 4222) and its corresponding protein models. Although whole-genome sequencing of the native isolate was precluded by resource and budgetary constraints during this study, we acknowledge the inherent genomic plasticity within the *B. thuringiensis* group. Therefore, these computational models are intended to serve as a highly representative theoretical framework rather than an exact strain-specific genomic map. However, because the core active-site scaffolds of these hydrolytic enzymes are highly conserved, this approach successfully illustrates the structural mechanisms and typical genomic architecture that fundamentally drive the powerful biocontrol and detoxification functions we functionally validated in our experimental assays. The whole-genome phylogenomic study using the TYGS platform, going beyond the resolution of single-gene markers, definitively established the identity of the isolate as belonging to the *B. thuringiensis* species (with 99% branch support), a critical step for distinguishing it from other closely related species (Meier-Kolthoff et al., 2013, 2022; Meier-Kolthoff and Göker, 2019). Annotating the reference genome using the RAST service revealed the common genetic basis for this antagonistic activity. It showed a multifunctional genome harboring a number of subsystems for sporulation and adaptation to stress, which are essential for the stability and persistence of a biocontrol agent in the field (Brettin et al., 2015). We emphasize that these genomic features are inferred (not sequenced) for our indigenous Egyptian isolate on the basis of this canonical genome. However, this comparative genomic analysis is highly corroborated by our *in vitro* biochemical studies, showing how this *B. thuringiensis* strain is inherently genetically programmed for the specific biocontrol, hydrolytic, and detoxification activities we have measured in our *in vitro* tests.

The bioactivity of the cell-free filtrate of *B. thuringiensis* increases notably after low-dose gamma irradiation (0.2 kGy), enhancing its antifungal activities and OTA degradation, with a non-monotonic, biphasic response curve in which activity is suboptimal at doses below 0.2 kGy and at least 99% lower at doses of 1 kGy or higher due to biomolecular damage. This bioenhancement effect at 0.2 kGy is likely caused by the mild oxidative effect exerted on biomolecules by reactive oxygen species (ROS) generated during water radiolysis, which induce slight structural changes in bioactive compounds, such as enzymes and cyclic lipopeptides, resulting in subtle refolding to better accommodate substrates in the active site and facilitate catalysis (Stanca et al., 2023). Higher doses (>1 kGy) irreversibly denature, aggregate, and break down the peptide bonds of proteins, abolishing their activities, as reported for high-dose (>5 kGy) irradiation (Li et al., 2025). Thus, longer irradiation times (during which these high doses are accumulated) ultimately destroy enzymatic functions because the proteins are subjected to severe oxidative stress and major structural degradation, demonstrating that irradiation time is critical to protein stability. Therefore, prolonged irradiation times, which correspond to these higher accumulated doses, ultimately abolish enzymatic functions by subjecting the proteins to excessive oxidative stress and catastrophic structural degradation, confirming that exposure time must be strictly limited to preserve protein stability. Enzymatic biotransformation is an effective method of detoxifying mycotoxins (Ding et al., 2023). This process mainly involves the cleavage of the OTA amide bond by carboxypeptidase, resulting in less toxic products. The action of carboxypeptidase on the OTA amide bond produces less harmful degradation products, including OTα and L-phenylalanine. Therefore, carboxypeptidase is essential for OTA degradation by modifying its chemical structure, transforming it into smaller, less toxic compounds (Ben Miri et al., 2024).

The current findings show that the greatly enhanced OTA detoxification at the low dose of 0.2 kGy is achieved by the cleavage of the amide bond between the L-phenylalanine and the isocoumarin backbone, leading to the formation of the less toxic metabolite OTα. In a crucial finding, the presence of this particular metabolite demonstrates that OTA degradation was caused by the active biocatalysis of the bacterial filtrate, rather than merely the suppression of fungal growth preventing the formation of OTA. This biotransformation capacity is further supported by the wheat grain experiment, where the biocatalytic filtrate was able to degrade OTA that had preformed during the 5 days of pre-incubation. Additionally, our data strongly suggest that the degradation of OTA is catalyzed by a carboxypeptidase, since the elevated OTα content correlated with a substantial increase in carboxypeptidase activity following irradiation (Ábrahám et al., 2025). However, applying cell-free filtrates can pose a bioprocess engineering problem due to a lack of stability and substrate entrapment. Future scale-up will need to address these limitations by developing and implementing novel delivery models, such as advanced immobilization techniques (Liu et al., 2024; Ábrahám et al., 2025). We demonstrate that sub-lethal gamma irradiation can be an effective bioprocessing strategy. This method helps in producing an improved biocontrol strain with enhanced antifungal activity and detoxification capabilities, providing a feasible avenue for use in agriculture and industry (Moradinezhad and Ranjbar, 2023; Liu et al., 2024).

Our analysis of the mechanism for the increased biocontrol activity of the cell-free filtrate of *B. thuringiensis* following gamma-ray irradiation at a low dose (0.2 kGy), which inhibits sterol biosynthesis and decomposes ochratoxin A (OTA), using Fourier-transform infrared spectroscopy (FTIR) and molecular techniques, revealed that the low dose of gamma rays causes molecular changes leading to a more active cell-free filtrate. FTIR analysis revealed a 2.32% increase in Amide I (1653.5 cm^−1^) and a 0.72% increase in Amide II (1525.5 cm^−1^) absorbance. It is important to note that the cell-free filtrate represents a secretome. Therefore, the detected changes of approximately 2% in the Amide I and Amide II bands reflect the general chemical response of the entire protein pool (secretome) and confirm that the 0.2 kGy radiation dose produces subtle, non-denaturing, bulk changes to the proteins. The specific carboxypeptidase assay serves as a readout of the effect of these global changes (Stanca et al., 2023). Most importantly, the sharp 5.82% increase in absorbance at 3454.5 cm^−1^ (O-H/N-H vibration) is likely due to the radiolysis of water and the generation of reactive oxygen species (ROS). This is not degradation, but rather the core driver of enhanced biocatalysis. Irradiation-induced ROS are responsible for specific amino acid side-chain oxidation (as modeled in our docking simulations) that affects the dynamics and electrostatic properties of the target active site, resulting in improved specific carboxypeptidase activity (18.06%) (Kumar and Barth, 2010; Tewari et al., 2023; Stanić et al., 2024). The suggested structural integrity of the cyclic lipopeptides, indicated by minuscule changes in the C=O stretching (1734.5 cm^−1^), reveals their role in the increased antifungal activity through fungal cell disruption (Neacsu et al., 2022; Stanca et al., 2023).

These computational results offer important insights into the potential biophysical underpinnings of the observed enzyme enhancement. However, it must be realized that gamma irradiation is a stochastic process. Radiation treatment at a dose of 0.2 kGy results in non-specific, random oxidative modifications, and not direct, targeted amino acid substitutions in the complex secretome. Thus, the modifications that were employed in our computational models, such as the hypothetical Asp142 to Arg substitution, are not exact radiolytic mutations that actually took place uniformly in the physical sample. Rather, since canonical molecular docking programs require discrete amino acid inputs, these specific mutations were only used as theoretical proxies. They are meant to qualitatively model the extreme regional electrostatic shifts and inversions that occur randomly in the protein backbone after being subjected to the random radiolytic oxidation, as demonstrated by our FTIR measurements. Through such radical electrostatic variations, this proof-of-concept simulation shows how some members within the stochastically oxidized pool of the enzyme might adopt highly optimal states. This accounts for the resultant macroscopic gain in the total carboxypeptidase activity *in vitro*, revealing how low-dose gamma radiation can be used as a way of physically tuning enzymes to supplement commonly used (Aly et al., 2017; Bian et al., 2022). Methionine-sulfoxide formation at Met140 results in the addition of a polar sulfoxide group that increases the rigidity of the region while forming new hydrogen bonds, which leads to an improved instability index and a preserved secondary structure (Marciniak and Bobrowski, 2022; Jabin et al., 2023). Tyr198 hydroxylation is responsible for the extra hydrogen bonding with OTA, as well as stabilizing *π*-π interactions with other aromatic amino acids, which is reflected in the 2.8% increase in overall aromatic content (Kinateder et al., 2023). In our model docking study, the hypothetical modification from Asp142 to Arg depicted charge reversals (representing extensive oxidative modifications) that could establish highly attractive interactions with carboxyl-rich substrate molecules, leading to improved binding affinities (Dickmann et al., 2004). These hypothetical modifications illustrate the modulatory effect of controlled gamma irradiation on enzyme structural integrity and activity through random radiolytic modifications. These macroscopic findings are consistent with recent research on radiation-induced physicochemical modifications, which shows that random ROS-induced oxidative hits can, on occasion, produce protein variants with optimal stability and efficacy (Hara, 2023; Ilderbayeva et al., 2025). Crucially, this abiotic improvement is distinct from the living stress response and regulatory hormesis seen when irradiating living cells.

Future work will focus on translating the current results into food safety applications. A key focus will be improving the activity of *B. thuringiensis* formulations on solid food matrices, such as cereals, through innovative delivery methods such as advanced immobilization techniques or preparation with food-grade carriers. Moreover, studies will focus on assessing the scalability, stability, and efficacy of irradiated filtrates under various storage and processing conditions. Furthermore, regulatory and biosafety considerations will be crucial aspects for certifying food safety. Finally, while the present computational models are strong theoretical predictors, we recognize the limitation of relying on *a priori* knowledge of structural radiolytic modifications. While this study assessed the gross effects of the cell-free secretome mixture, subsequent research must involve targeted downstream purification of the native carboxypeptidase to link this specific enzyme to OTA degradation in a biochemical, causal manner. As such, future research should employ direct structural and analytical methods, such as circular dichroism (CD) spectroscopy and targeted mass spectrometry (LC–MS/MS). Such techniques are essential to unequivocally define the exact molecular changes induced by radiation, confirm secondary structure changes, and experimentally validate the hypothetical proof-of-concept docking models proposed in this study.

## Conclusion

This research shows that sub-lethal gamma radiation can be used as a means of enhancing the enzymatic system of *B. thuringiensis*. Through the structural and functional optimization of carboxypeptidase, this results in a much faster biotransformation of toxic ochratoxin A into less toxic products. This represents an enzyme-based biotechnological development for safe mycotoxin detoxification in agricultural and environmental settings, demonstrating a statistically significant increase in the suppression of *A. niger* growth, as well as the specific enzymatic conversion of OTA to the less toxic OTα. These detoxification findings were complemented by *in silico* molecular docking simulations, providing insight into the plausible mechanisms of this enhanced bioactivity. These computational findings provided strong, convergent evidence implicating carboxypeptidase as the key enzyme responsible for the enhanced detoxification, demonstrating that the 0.2 kGy dose optimally improved both its structural stability and OTA binding potential. These findings validate low-dose gamma irradiation as a promising strategy to enhance the potential of the investigated *B. thuringiensis* isolate as a biocontrol agent. This bio-enhanced strain can be utilized in the development of promising commercial bioproducts for controlling mycotoxin issues and enhancing food quality and safety.

## Data Availability

The datasets presented in this study can be found in online repositories. The names of the repository/repositories and accession number(s) can be found in the article/supplementary material.

## Footnotes

1
https://tygs.dsmz.de

2
https://rast.nmpdr.org

3 Available at https://proksee.ca.

## References

[ref1] Abbasi E. (2025). Emerging strategies in food safety: innovations in microbial risk mitigation, biopreservation, and sustainable packaging technologies. Int. J. Food Sci. Technol. 60:vvaf175. doi: 10.1093/ijfood/vvaf175

[ref2] Ábrahám R. Baka E. Al-Nussairawi M. Táncsics A. Farkas M. Nagy I. (2025). Molecular insights into ochratoxin a biodegradation. Biol. Futur. 76, 315–328. doi: 10.1007/s42977-025-00258-2, 40374978

[ref3] Aly E. E. Khalil N. M. El-Hamid G. A. Abo-El-Soued M. A. Mostafa H. S. (2017). Mutagenic effect of gamma irradiation on phenotype and enzyme activities in *Aspergillus niger*. Egypt. J. Bot. 56, 507–526. doi: 10.21608/ejbo.2017.1148

[ref4] Asif T. Javed U. Zafar S. B. Ansari A. Ul Qader S. A. Aman A. (2020). Bioconversion of colloidal chitin using novel chitinase from *Glutamicibacter uratoxydans* exhibiting anti-fungal potential by hydrolyzing chitin within fungal cell wall. Waste Biomass Valoriz. 11, 4129–4143. doi: 10.1007/s12649-019-00746-2

[ref5] Awaad H. A. Negm A. M. Abu-hashim M. (2021). “Update, conclusions, and recommendations of ‘mitigating environmental stresses for agricultural sustainability in Egypt’,” in Mitigating Environmental Stresses for Agricultural Sustainability in Egypt, (Cham: Springer), 561–590. doi: 10.1007/978-3-030-64323-2_21

[ref6] Badawy I. H. Hmed A. A. Sofy M. R. Al-Mokadem A. Z. (2022). Alleviation of cadmium and nickel toxicity and phyto-stimulation of tomato plant L. by endophytic *Micrococcus luteus* and *Enterobacter cloacae*. Plants 11:2018. doi: 10.3390/plants11152018, 35956496 PMC9370581

[ref7] Balendres M. A. O. Karlovsky P. Cumagun C. J. R. (2019). Mycotoxigenic fungi and mycotoxins in agricultural crop commodities in the Philippines: a review. Foods 8:249. doi: 10.3390/foods8070249, 31288486 PMC6678526

[ref8] Ben Miri Y. Benabdallah A. Chentir I. Djenane D. Luvisi A. De Bellis L. (2024). Comprehensive insights into ochratoxin a: occurrence, analysis, and control strategies. Foods 13:1184. doi: 10.3390/foods13081184, 38672856 PMC11049263

[ref9] Bian Z. Tu Z. Wang H. Hu Y. Liu G. (2022). Investigation of the mechanism of 60Co gamma-ray irradiation-stimulated oxidation enhancing the antigenicity of ovalbumin by high-resolution mass spectrometry. J. Agric. Food Chem. 70, 9477–9488. doi: 10.1021/acs.jafc.2c03911, 35881501

[ref10] Brettin T. Davis J. J. Disz T. Edwards R. A. Gerdes S. Olsen G. J. . (2015). RASTtk: a modular and extensible implementation of the RAST algorithm for building custom annotation pipelines and annotating batches of genomes. Sci. Rep. 5:8365. doi: 10.1038/srep08365, 25666585 PMC4322359

[ref11] Calicis C. Christiaens R. Loquet N. Simon M. Collin S. (2025). Measuring the carboxypeptidase and γ-glutamyltranspeptidase activities of lager and ale yeasts to assess their impact on the release of odorant polyfunctional thiols through fermentation. Molecules 30:2491. doi: 10.3390/molecules30122491, 40572456 PMC12196205

[ref12] Camacho C. Coulouris G. Avagyan V. Ma N. Papadopoulos J. Bealer K. . (2009). BLAST+: architecture and applications. BMC Bioinformatics 10:421. doi: 10.1186/1471-2105-10-421, 20003500 PMC2803857

[ref13] Chandravanshi K. Singh R. Kumar A. Bhange G. N. Kumar A. Makde R. D. (2024). Structural adaptations for carboxypeptidase activity in putative s9 acylaminoacyl peptidase from *Bacillus subtilis*. Int. J. Biol. Macromol. 282:136734. doi: 10.1016/j.ijbiomac.2024.136734, 39433196

[ref14] Davies M. J. (2016). Protein oxidation and peroxidation. Biochem. J. 473, 805–825. doi: 10.1042/BJ20151227, 27026395 PMC4819570

[ref15] Demasi M. Augusto O. Bechara E. J. H. Bicev R. N. Cerqueira F. M. da Cunha F. M. . (2021). Oxidative modification of proteins: from damage to catalysis, signaling, and beyond. Antioxid. Redox Signal. 35, 1016–1080. doi: 10.1089/ars.2020.8176, 33726509

[ref16] Dickmann L. J. Locuson C. W. Jones J. P. Rettie A. E. (2004). Differential roles of arg97, asp293, and arg108 in enzyme stability and substrate specificity of CYP2C9. Mol. Pharmacol. 65, 842–850. doi: 10.1124/mol.65.4.842, 15044613

[ref17] Ding L. Han M. Wang X. Guo Y. (2023). Ochratoxin A: overview of prevention, removal, and detoxification methods. Toxins (Basel) 15:565. doi: 10.3390/toxins15090565, 37755991 PMC10534725

[ref18] Esuola C. O. Ortega-Beltran A. (2025). Fumonisin and ochratoxin-producing strains of *aspergillus* section nigri are associated with onion (*Allium cepa* L.) bulbs sold in markets in Southwest Nigeria. Front. Fungal Biol. 6:1563824. doi: 10.3389/ffunb.2025.1563824, 40337159 PMC12056510

[ref19] Fakhri Y. Mehri F. Ranaei V. Pilevar Z. Soleimani F. Nasiri R. (2025). The prevalence and concentration of mycotoxins (aflatoxins, deoxynivalenol, zearalenone, and ochratoxin A) in domestic bird eggs: a global systematic review, meta-analysis, and probabilistic risk assessment. J. Food Prot. 88:100600. doi: 10.1016/j.jfp.2025.100600, 40819745

[ref20] Gherbawy Y. A. Hamza L. H. A. Maghraby T. A. Shebany Y. M. Abdelwahab S. F. El-Dawy E. G. A. M. (2025). Characterization of *aspergillus tubingensis* from grains in upper Egypt and their potential for ochratoxins production. Food Biotechnol. 39, 28–50. doi: 10.1080/08905436.2025.2458242

[ref21] Grant J. R. Enns E. Marinier E. Mandal A. Herman E. K. Chen C. . (2023). Proksee: in-depth characterization and visualization of bacterial genomes. Nucleic Acids Res. 51, W484–W492. doi: 10.1093/nar/gkad326, 37140037 PMC10320063

[ref22] Gull Naz A. A. A. Ali T. Nawaz M. Iqbal S. (2023). Molecular characterization of ochratoxin A producing indigenous *aspergillus* strains from poultry feed in Pakistan. Sains Malaysiana 52, 821–835. doi: 10.17576/jsm-2023-5203-11

[ref23] Gurikar C. Shivaprasad D. P. Sabillón L. Gowda N. A. N. Siliveru K. (2023). Impact of mycotoxins and their metabolites associated with food grains. Grain Oil Sci. Technol. 6, 1–9. doi: 10.1016/j.gaost.2022.10.001

[ref24] Hara M. (2023). Effects of ionizing radiation on biopolymers for applications as biomaterials. Biomed. Mater. Devices 1, 587–604. doi: 10.1007/s44174-022-00049-6

[ref25] Ilderbayeva G. Rakhyzhanova S. Utegenova A. Salkhozhayeva G. Ilderbayev O. (2025). Combined effect of gamma radiation and heavy metals on some living organisms. Biol. Trace Elem. Res. 203, 1764–1775. doi: 10.1007/s12011-024-04272-8, 38907828

[ref26] Ismaiel A. A. Mohamed H. H. El-Sayed M. T. (2023). Biodegradation of ochratoxin a by endophytic *Trichoderma koningii* strains. World J. Microbiol. Biotechnol. 39:53. doi: 10.1007/s11274-022-03491-2, 36564607 PMC9789014

[ref27] Jabin T. Biswas S. Islam S. Sarker S. Afroze M. Paul G. K. (2023). Effects of gamma-radiation on microbial, nutritional, and functional properties of katimon mango peels: a combined biochemical and *in silico* studies. Heliyon 9:e21556. doi: 10.1016/j.heliyon.2023.e21556, 38027912 PMC10665690

[ref28] Jayaram M. Nagao H. (2018). Potato dextrose agar with rose-bengal and chloramphenicol: a new culture medium to isolate pathogenic *Exophiala dermatitidis* from the environment. Klimik J. 31, 11–15. doi: 10.5152/kd.2018.05

[ref29] Kinateder T. Drexler L. Straub K. Sterner R. (2023). Experimental and computational analysis of the ancestry of an evolutionary young enzyme from histidine biosynthesis. Protein Sci. 32:E4536. 10.1002/pro.4536 PMC9798254 36502290

[ref30] Kreft Ł. Botzki A. Coppens F. Vandepoele K. Van Bel M. (2017). PhyD3: a phylogenetic tree viewer with extended phyloXML support for functional genomics data visualization. Bioinformatics 33, 2946–2947. doi: 10.1093/bioinformatics/btx324 28525531

[ref31] Kumar S. Barth A. (2010). Following enzyme activity with infrared spectroscopy. Sensors 10, 2626–2637. doi: 10.3390/s100402626, 22319264 PMC3274194

[ref32] Lagesen K. Hallin P. Rødland E. A. Stærfeldt H.-H. Rognes T. Ussery D. W. (2007). RNAmmer: consistent and rapid annotation of ribosomal rna genes. Nucleic Acids Res. 35, 3100–3108. doi: 10.1093/nar/gkm160, 17452365 PMC1888812

[ref33] Lefort V. Desper R. Gascuel O. (2015). FastME 2.0: a comprehensive, accurate, and fast distance-based phylogeny inference program. Mol. Biol. Evol. 32, 2798–2800. doi: 10.1093/molbev/msv150, 26130081 PMC4576710

[ref34] Li H. Li C. Shoaib M. Zhang W. Murugesan A. (2025). Advances in non-thermal processing of meat and monitoring meat protein gels through vibrational spectroscopy. Foods 14:1929. doi: 10.3390/foods14111929, 40509457 PMC12155568

[ref35] Li X. J. Yao C. X. Qiu R. Bai J. K. Liu C. Chen Y. G. (2023). Isolation, identification, and evaluation of the biocontrol potential of a *Bacillus velezensis* strain against tobacco root rot caused by *fusarium oxysporum*. J. Appl. Microbiol. 134:lxac049. doi: 10.1093/jambio/lxac049, 36626796

[ref36] Liu M. Zhang X. Luan H. Zhang Y. Xu W. Feng W. (2024). Bioenzymatic detoxification of mycotoxins. Front. Microbiol. 15:1434987. doi: 10.3389/fmicb.2024.1434987, 39091297 PMC11291262

[ref37] Marciniak B. Bobrowski K. (2022). Photo-and radiation-induced one-electron oxidation of methionine in various structural environments studied by time-resolved techniques. Molecules 27:1028. doi: 10.3390/molecules27031028, 35164293 PMC8915190

[ref38] Meier-Kolthoff J. P. Auch A. F. Klenk H.-P. Göker M. (2013). Genome sequence-based species delimitation with confidence intervals and improved distance functions. BMC Bioinformatics 14:60. doi: 10.1186/1471-2105-14-60, 23432962 PMC3665452

[ref39] Meier-Kolthoff J. P. Carbasse J. S. Peinado-Olarte R. L. Göker M. (2022). TYGS and LPSN: a database tandem for fast and reliable genome-based classification and nomenclature of prokaryotes. Nucleic Acids Res. 50, D801–D807. doi: 10.1093/nar/gkab902, 34634793 PMC8728197

[ref40] Meier-Kolthoff J. P. Göker M. (2019). TYGS is an automated high-throughput platform for state-of-the-art genome-based taxonomy. Nat. Commun. 10:2182. doi: 10.1038/s41467-019-10210-3, 31097708 PMC6522516

[ref41] Minato T. Nirasawa S. Sato T. Yamaguchi T. Hoshizaki M. Inagaki T. (2020). B38-CAP is a bacteria-derived ace2-like enzyme that suppresses hypertension and cardiac dysfunction. Nat. Commun. 11:1058. doi: 10.1038/s41467-020-14867-z, 32103002 PMC7044196

[ref42] Mohamed A. M. Sayed A. A Abd El-Aziz F. Abd-Elmonsef Mahmoud G. (2023). Screening of bacterial isolates for protease production with special reference to molecular identification of highly producer strains. J. Appl. Mol. Biol. 1, 12–30. doi: 10.21608/jamb.2023.219174.1007

[ref43] Moradinezhad F. Ranjbar A. (2023). Advances in postharvest diseases management of fruits and vegetables: a review. Horticulturae 9:1099. doi: 10.3390/horticulturae9101099

[ref44] Mufida D. R. A. Putra I. P. Nawangsih A. A. Krishanti N. P. R. A. Wahyudi A. T. (2024). Glucanase enzyme activity from rhizospheric *Streptomyces* spp. inhibit growth and damage the cell wall of *Fusarium oxysporum*. Rhizosphere 32:100991. doi: 10.1016/j.rhisph.2024.100991

[ref45] Neacsu A. Gheorghe D. Tecuceanu V. Perişanu Ş. (2022). Investigation of thermochemical features of gamma irradiated tryptophan stereoisomers. J. Mex. Chem. Soc. 66, 42–56. doi: 10.29356/jmcs.v66i1.1627

[ref46] Nguyen T. Chen X. Ma L. Feng Y. (2024). Mycotoxin biodegradation by *Bacillus* bacteria—a review. Toxins (Basel) 16:478. doi: 10.3390/toxins16110478, 39591233 PMC11598562

[ref47] Okoye C. O. Ezenwanne B. C. Olalowo O. O. Ajanwachukwu O. J. Chukwudozie K. I. (2025). Microbial-mycotoxin interactions in food: a review of ecotoxicological implications and omics approaches for understanding detoxification mechanisms. Food Microbiol.:104955. doi: 10.1016/j.fm.2025.104955 41344758

[ref48] Ondov B. D. Treangen T. J. Melsted P. Mallonee A. B. Bergman N. H. Koren S. . (2016). Mash: fast genome and metagenome distance estimation using minhash. Genome Biol. 17:132. doi: 10.1186/s13059-016-0997-x, 27323842 PMC4915045

[ref49] Osman G. E. H. El-Ghareeb D. Already R. Assaeedi A. S. A. Organji S. R. Abulreesh H. H. (2015). Bioinsecticide *Bacillus thuringiensis* a comprehensive review. Egypt. J. Biol. Pest Control 25, 271–288. doi: 10.5555/20153293608

[ref50] Radomska K. Wolszczak M. (2023). Influence of ionizing radiation on spontaneously formed aggregates in proteins or enzymes solutions. Pharmaceutics 15:1367. doi: 10.3390/pharmaceutics15051367, 37242609 PMC10221975

[ref51] Ramona Y. A. N. (2021). Growth inhibition of fungal plant pathogens by antagonist bacteria using dual culture assays. Biotropia 28, 231–238. doi: 10.11598/btb.2021.28.3.1344

[ref52] Rawat R. Sharma M. Singh P. (2024). “Biotechnology and its position in the mitigation of microbial problems in the food industry,” in Microbial Biotechnology in the Food Industry: Advances, Challenges, and Potential Solutions, (Canada: Springer), 103–127.

[ref53] Rimsa V. Eadsforth T. C. Joosten R. P. Hunter W. N. (2014). High-resolution structure of the m14-type cytosolic carboxypeptidase from *Burkholderia cenocepacia* refined exploiting pdb_redo strategies. Biol. Crystallogr. 70, 279–289. doi: 10.1107/S1399004713026801, 24531462 PMC3940198

[ref54] Riseh R. S. Vatankhah M. Hassanisaadi M. Ait Barka E. (2024). Unveiling the role of hydrolytic enzymes from soil biocontrol bacteria in sustainable phytopathogen management. Front. Biosci. 29:105. doi: 10.31083/j.fbl2903105, 38538262

[ref55] Rostami M. Ghorbani A. Shahbazi S. (2024). Gamma radiation-induced enhancement of biocontrol agents for plant disease management. Curr. Res. Microb. Sci. 7:100308. doi: 10.1016/j.crmicr.2024.100308, 39620098 PMC11605434

[ref56] Rustagi V. Gupta S. R. R. Bajaj M. Singh A. Singh I. K. (2023). PepAnalyzer: predicting peptide properties using its sequence. Amino Acids 55, 1371–1379. doi: 10.1007/s00726-023-03317-x, 37668712

[ref57] Sa’diyah W. Hashimoto A. Okada G. Ohkuma M. (2021). Notes on some interesting sporocarp-inhabiting fungi isolated from xylarialean fungi in Japan. Diversity 13:574. doi: 10.3390/d13110574

[ref58] Stanca M. Gaidau C. Zaharescu T. Balan G.-A. Matei I. Precupas A. (2023). Physico-chemical changes induced by gamma irradiation on some structural protein extracts. Biomolecules 13:774. doi: 10.3390/biom13050774, 37238645 PMC10216533

[ref59] Stanić M. Jevtović M. Kovačević S. Dimitrijević M. Danilović Luković J. McIntosh O. A. (2024). Low-dose ionizing radiation generates a hormetic response to modify lipid metabolism in *Chlorella sorokiniana*. Commun. Biol. 7:821. doi: 10.1038/s42003-024-06526-6, 38969726 PMC11226653

[ref60] Tang Y. Wang Z. Feng H. Wang L. (2026). Evaluation of the inhibitory effect of the cell-free fermentation filtrate of *Bacillus atrophaeus* YL84 on *Fusarium oxysporum* f. sp. vasinfectum and analysis of its metabolic products. Front. Microbiol. 17:1761389. doi: 10.3389/fmicb.2026.1761389, 41657909 PMC12872879

[ref61] Tewari K. Kesawat M. S. Kumar V. Maheshwari C. Krishnan V. Narwal S. (2023). “Role of gamma irradiation in enhancement of nutrition and flavor quality of soybean,” in Gamma Rays-Current Insights, (India: IntechOpen).

[ref62] Veras F. F. Silveira R. D. Welke J. E. (2023). *Bacillus* spp. as a strategy to control fungi and mycotoxins in food. Curr. Opin. Food Sci. 52:101068. doi: 10.1016/j.cofs.2023.101068

[ref63] Vignesh S. Rajadurai G. Raghu R. Balakrishnan N. Jayakanthan M. Mohankumar S. (2024). Potential applications of *Bacillus thuringiensis* berliner in agriculture, medicine and environment. Plant Sci. Today 11:3977. doi: 10.14719/pst.3977

[ref64] Wang J. Sufar E. K. Bernhoft A. Seal C. Rempelos L. Hasanaliyeva G. (2024). Mycotoxin contamination in organic and conventional cereal grain and products: a systematic literature review and meta-analysis. Compr. Rev. Food Sci. Food Saf. 23:e13363. doi: 10.1111/1541-4337.13363, 38720588

[ref65] Xiong L. Peng M. Zhao M. Liang Z. (2020). Truncated expression of a carboxypeptidase a from bovine improves its enzymatic properties and detoxification efficiency of ochratoxin a. Toxins (Basel) 12:680. doi: 10.3390/toxins12110680, 33137913 PMC7692142

[ref66] Yassein A. S. Elamary R. B. (2021). Efficacy of soil *Paraburkholderia fungorum* and *Bacillus subtilis* on the inhibition of *Aspergillus niger* growth and its ochratoxins production. Egypt. J. Bot. 61, 319–334. doi: 10.21608/ejbo.2021.68481.1656

[ref67] Yi Y. Hou Z. Yang Q. Cui L. Lu H. Li R. (2023). Antimicrobial mechanism and biocontrol effect of *Bacillus cereus* XZ30-2 on *Aspergillus niger*. Qual. Assur. Saf. Crop. Foods 15, 77–88. doi: 10.15586/qas.v15i4.1379

[ref68] Yuan X.-L. van der Kaaij R. M. van den Hondel C. A. Punt P. J. van der Maarel M. J. E. C. Dijkhuizen L. . (2008). *Aspergillus niger* genome-wide analysis reveals a large number of novel alpha-glucan acting enzymes with unexpected expression profiles. Mol. Gen. Genomics. 279, 545–561. doi: 10.1007/s00438-008-0332-7, 18320228 PMC2413074

[ref69] Zhang D. Huang K. Ye C. Zou D. Liu D. Wei X. (2024). Enhancing biological control of apple rot: unveiling the antifungal potential and mechanism of *Bacillus amyloliquefaciens* HZ-12′ s lipopeptide. Sci. Hortic. (Amsterdam) 325:112704. doi: 10.1016/j.scienta.2023.112704

[ref70] Zhao M. Liu D. Liang Z. Huang K. Wu X. (2022). Antagonistic activity of *Bacillus subtilis* CW14 and its β-glucanase against *aspergillus ochraceus*. Food Control 131:108475. doi: 10.1016/j.foodcont.2021.108475

[ref71] Zou D. Ji J. Ye Y. Yang Y. Yu J. Wang M. (2022). Degradation of ochratoxin a by a uv-mutated *Aspergillus niger* strain. Toxins 14:343. doi: 10.3390/toxins14050343, 35622590 PMC9146908

